# Survey of background scattering from materials found in small-angle neutron scattering

**DOI:** 10.1107/S1600576715010729

**Published:** 2015-06-27

**Authors:** J. G. Barker, D. F. R. Mildner

**Affiliations:** a NIST Center for Neutron Research, National Institute of Standards and Technology, Gaithersburg, Maryland 20899, USA

**Keywords:** small-angle neutron scattering, background scattering, multiple scattering, phonon scattering, time-of-flight measurements

## Abstract

Measurements and calculations of beam attenuation and background scattering for common materials placed in a neutron beam are presented over the temperature range of 300–700 K. Time-of-flight (TOF) measurements have also been made, to determine the fraction of the background that is either inelastic or quasi-elastic scattering as measured with a ^3^He detector. Other background sources considered include double Bragg diffraction from windows or samples, scattering from gases, and phonon scattering from solids. Background from the residual air in detector vacuum vessels and scattering from the ^3^He detector dome are presented. The thickness dependence of the multiple scattering correction for forward scattering from water is calculated. Inelastic phonon background scattering at small angles for crystalline solids is both modeled and compared with measurements. Methods of maximizing the signal-to-noise ratio by material selection, choice of sample thickness and wavelength, removal of inelastic background by TOF or Be filters, and removal of spin-flip scattering with polarized beam analysis are discussed.

## Abstract 2

The paper describes quantitative methods for determining the background scattering from materials, including gases, liquids and solids, found in small-angle neutron scattering measurements. By understanding the dependence of the background on material type, thickness, wavelength and temperature, the investigator can design experiments that minimize the background scattering and thus improve the statistical accuracy of the background-corrected data.

## 1. Introduction   

### 1.1. Materials   

In the design of neutron scattering instrumentation, materials need to be placed in the neutron beam path to act as windows, filters, collimators or monochromators. Careful consideration is needed in choosing optimal materials that reduce the interaction with the neutron beam to minimize both beam attenuation and background on the detector. The materials may be broken into several categories. (1) Gases: on the basis of instrument design, the beam path may be evacuated or filled with air or some other gases with smaller scattering cross section such as helium or argon. (2) Vessel windows: to separate different gas-filled or evacuated vessels in the beam path, windows having sufficient strength and thickness to support any pressure differential between individual sections are used. For low attenuation, aluminium and magnesium alloys are typically used. The higher strength and fracture toughness of the metal alloys allow windows as thin as 0.1 mm to support 10^5^ Pa of pressure. In cases requiring lower background, thicker single-crystal silicon (opaque to light) or sapphire (transparent to light) is used. (3) Sample environment: lower-cost windows that can handle corrosive environments are often made from quartz (amorphous SiO_2_). At higher temperatures, heat reflection shields in the form of aluminium or niobium foils are often used. (4) Crystal monochomators: common monochromator materials in the beam are mosaic or perfect single crystals of graphite, silicon, germanium or copper. (5) Neutron filters: Materials used to filter fast, epithermal or thermal neutrons are polycrystalline Be or BeO, mosaic crystals of graphite, single crystals of Si, sapphire, MgF_2_, or MgO. Polycrystalline blocks of bismuth with grains several centimetres in size are used for gamma-ray filtering. (6) Sample solvents: many organic liquids are used in samples for small-angle neutron scattering (SANS) experiments. By using deuterated organic solvents, the scattering contrast is often enhanced, which increases the SANS intensity while both the beam attenuation and background scattering are reduced.

We review information from the literature and present new measurements on common materials placed in the beam. We use these results to calculate the beam attenuation and the background scattering dependence on temperature for the typical SANS scattering angle range (0 ≤ θ ≤ 30°). TOF measurements have also been made, to determine the fraction of the background scattering that is inelastic *versus* quasi-elastic. Further background reduction is achievable by pulsing the beam with a disc chopper and discriminating the inelastic scattering events. We also discuss methods to mitigate background scattering from magnetic materials, dislocations and Bragg scattering.

### 1.2. Background scattering   

SANS instruments typically use either rectangular or circular source and sample apertures to collimate a beam. The range of neutron wavelengths is chosen either by a helical velocity selector at steady (nuclear reactor) sources or using time-of-flight (TOF) measurements at pulsed spallation neutron sources. The sample is placed directly after the sample aperture. A two-dimensional detector is typically placed in a large vacuum vessel after the sample, the distance from sample to detector being *L*_2_. When the measured scattering on the two-dimensional detector is axially symmetric, the data are grouped into annuli having mean radius *R_i_* and width Δ*R*. The scattering vector magnitude is defined as *q_i_* = (4π/λ_0_) ×sin(θ*_i_*/2) ≃ 2πθ*_i_*/λ_0_, where λ_0_ is the mean neutron wavelength and the scattering angle θ_*i*_ = tan^−1^(*R_i_*/*L*_2_). In TOF instruments, the scattering vectors *q_i_* are further grouped according to the wavelength associated with a particular time bin.

The instrument background is subtracted from the sample scattering by
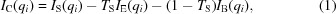
where the subscripts stand for C = corrected, S = sample, E = empty and B = blocked, *I* is the intensity, and *T*_S_ is the sample transmission. The sample scattering is typically obtained with the sample in a holder. The sample normally has slab geometry of constant thickness. The blocked scattering is obtained by placing a neutron absorber such as cadmium foil in place of the slab sample. The empty scattering is obtained by removing the sample from the holder. For dilute solutions, the solvent can be kept in the holder for the empty measurement. The sample transmission *T*_S_ is determined by measuring the ratio of the beam current with the sample to that of the empty or solvent-containing holder. For a detailed description of refinements to the above simplified background correction, see Brûlet *et al.* (2007 ▸). The above intensities are usually normalized to a fixed number of neutron counts in a low-efficiency beam monitor to correct for fluctuations in source strength.

The absolute macroscopic scattering cross section is determined by
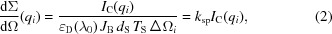
where ∊_D_ is the detector efficiency, which depends upon the neutron wavelength λ_0_, *J*_B_ is the beam current incident on the sample, *d*_S_ is the sample thickness and ΔΩ*_i_* ≃ 2πΔ*R* *R_i_*/*L*_2_^2^ is the solid angle subtended by the *i*th annulus on the detector. For TOF measurements, each time bin *j* has a separate value for ∊_D_(λ*_j_*)*J*_B,*j*_. The quantity ∊_D_ *J*_B_ is determined either by measuring separately the scattering from a known standard sample, measuring the beam current directly with the two-dimensional detector after attenuation by a known factor *A*_f_, or by using a calibrated low-efficiency beam monitor in front of the beamstop (Heenan *et al.*, 1997 ▸).

The variance *V*, equal to the square of the standard deviation σ (*V* = σ^2^), and thus the uncertainty in a measurement of the absolute cross section can be determined from separate measurements from the sample, empty and beam-blocked runs by
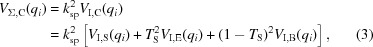
where subscript Σ denotes intensity in absolute units dΣ/dΩ, and subscript I the scattering intensity in arbitrary units but normalized to monitor count. If the monitor count rate is constant during the separate measurements, the intensities *I* will be proportional to the count rates *C* (*I* = *kC*). All uncertainties given in this paper represent one standard deviation. If the uncertainty is due solely to random counting statistics where the variance is equal to the total counts *V* = *tC* and where *t* is the counting time, the variances in intensity can be estimated simply as
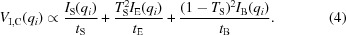
For an experiment, the separate counting times *t*_S_, *t*_E_ and *t*_B_ are typically chosen on the basis of the separate count rates and the number of samples. For cases where the sample scattering is weak compared to the empty background, *I*_C_ << *T*_S_ *I*_E_, the variance will also be dominated by the empty background in both the sample and empty runs. Counting statistics dictates that, if all the counting times are increased equally by a factor of four, the standard deviation of the measurement will be reduced by a factor of two. However, if the empty background is dominant, reducing the empty background count rate by a factor of four also reduces the deviation by a factor of two for the same counting time. This paper determines how the background corrections *I*_E_ and *I*_B_ are affected by placing different materials in the beam, so that by informed choices of materials the instrument background may be reduced in some situations, thus improving the precision of measurements.

The background scattering component at larger angles (θ ≥ 5°), particularly from hydrogenous materials, has a large fraction of inelastic scattering. Yet, the small-angle scattering (SAS) after the background correction as used in SAS measurements is typically assumed to be quasi-elastic; the scattering event changes the neutron energy only by a small amount by movement of macromolecules in solution. In solid samples the SAS can be viewed as being elastic. Typically, the background-corrected intensity still contains an additional sample scattering background component that has both quasi-elastic and inelastic components. For cold neutrons, having wavelength λ_0_ > 1.8 Å, the inelastic scattering from samples at room temperature on average increases the energy (up scatters) and thus reduces the wavelength. Since the efficiency of the detector often depends upon the wavelength and is typically lower than ∊_D_(λ_0_) at shorter wavelengths, the contribution of the inelastic background is reduced from the true absolute cross section by the ratio ∊_D_(λ)/∊_D_(λ_0_). This distortion in the observed cross section from differences in detector efficiency with neutron energy is discussed later with respect to organic liquids.

## 2. Scattering from gases   

### 2.1. Theory and calculation   

Typical gases encountered in SANS experiments and instrumentation are ambient air, nitrogen, helium or argon. To minimize the attenuation of the beam and the background scattering on the detector from gases located downstream from the source aperture, the beam path is typically evacuated except for a small area around the sample location. The vacuum chambers must be constructed to withstand ambient pressure and have suitable thin windows to transmit the neutron beam. These windows must be both strong enough to withstand the pressure differential and yet thin to produce minimal background scattering and beam attenuation. By using argon or helium at or near ambient pressure, the pressure difference between the inside and outside of the chamber can be greatly reduced. This enables the chamber to be designed with much thinner walls and at reduced cost. For example, the TPA SANS instrument at Saclay, France, uses helium at ambient pressure instead of vacuum in a detector vessel (Brûlet *et al.*, 2008 ▸). To simplify access to the sample and ancillary equipment, the sample area is usually in ambient air, with windows for the incident and scattered beams.

Absorption from the gases can be calculated from the dependence on the absorption cross section, which varies inversely with the neutron velocity. The absorption cross section for a gas is
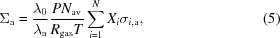
where λ_a_ = 1.798 Å {corresponding to a neutron velocity of 2200 ms^−1^, which is the mean wavelength for a Maxwellian distribution of neutron energies moderated at room temperature, at which absorption cross sections are typically tabulated [such as by Sears (1986 ▸, 1992 ▸)]}, *P* is the gas pressure, *T* is the gas temperature, *N*_av_ = 6.022 × 10^23^ mol^−1^ is Avogadro’s number, *R*_gas_ = 8.314 m^3^ Pa K^−1^ mol^−1^ is the ideal gas constant, *X_i_* is the mole fraction of molecule *i* and σ_*i*,a_ is the absorption cross section per molecule *i* at wavelength λ_a_.

The dependence of the total scattering cross section from gasses upon temperature *T* and wavelength λ can be estimated according to the formalism originally developed by Sachs & Teller (1941 ▸):
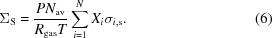
Here, the neutron wavelength and gas temperature dependence of the *i*th molecular gas cross section σ_*i*,s_ is calculated by
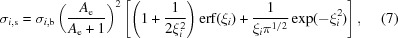
where *A*_e_ is the effective mass of molecule *i* in atomic mass units but altered to account for rotational weighting of the different atoms, and erf() is the error function. The effect of the gas temperature *T* and neutron wavelength λ are accounted for within the factor
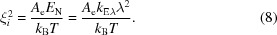
σ_*i*,b_ is the bound scattering cross section for the *i*th molecule, *k*_B_ = 0.08617 meV K^−1^ is Boltzmann’s constant, *E*_N_ is the neutron energy and *k*_Eλ_ = 81.81 meV Å^−2^ is a conversion constant from neutron energy to neutron wavelength.

**Figure 1:**
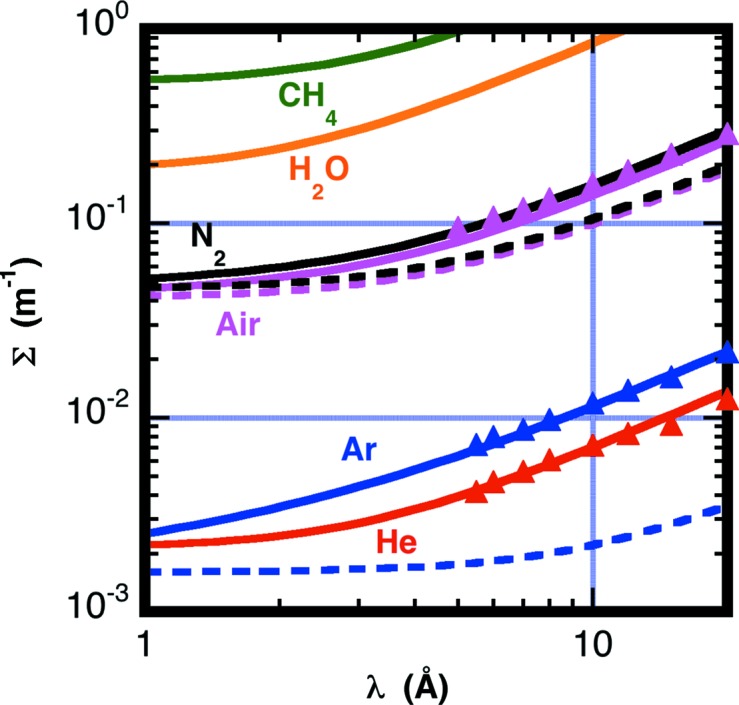
Calculated total (Σ_a_ + Σ_S_) cross sections, shown as solid lines, and scattering (Σ_S_) cross sections, shown as dashed lines, for five gases and air *versus* neutron wavelength at temperature *T* = 298 K and pressure *P* = 1 × 10^5^ Pa. The air composition assumes a water content corresponding to a dew point of *T*_d_ = 283 K. (Magenta = air, He = red, Ar = blue, N_2_ = black, H_2_O = orange and CH_4_ = green.) Solid symbols correspond to measured total cross sections.

Using equations (5)–(8) [Disp-formula fd7] [Disp-formula fd8] and the cross sections listed in Table 1 ▸, Fig. 1 ▸ shows both the calculated total cross sections (Σ_a_ + Σ_S_) shown as solid lines and the scattering cross sections (Σ_S_) shown as dashed lines for five pure gases and air as a function of neutron wavelength at typical ambient conditions: *T* = 298 K and *P* = 1 × 10^5^ Pa. Air is assumed to have a water content corresponding to a dew point of *T*_d_ = 283 K.

To verify the accuracy of the given expressions, the transmissions of air, helium and argon gas have been measured. The 13.3 m-long pre-sample vacuum vessel of the NG7 SANS instrument (Glinka *et al.*, 1998 ▸) was filled with the gases, and measurements were made at several neutron wavelengths. The solid symbols in Fig. 1 ▸ correspond to the measured total cross section of the three gases. The measured total cross sections for air are 10–15% higher than the calculated values in the wavelength range 5 ≤ λ_0_ ≤ 20 Å, possibly because the water content is higher than that stated in Table 1 ▸. The humidity was not determined during the measurements. The measurements for argon and helium are in good agreement with the calculation.

Note that for minimum beam attenuation helium has a smaller total cross section than argon. From the calculated values, the scattering and absorption from helium gas is equivalent to 5.0% of air. Replacing an air path with helium thus lowers the beam attenuation by a factor of 20, though replacing helium with argon can reduce the scattering background further. The total cross section of argon is a factor 1.6 higher than that of helium, resulting in higher beam attenuation; the scattering cross section of argon is only 0.31 of that of helium, thus reducing the scattering by a further factor of three from helium, or a factor of 46 with respect to air. Argon will activate from neutron radiation with a 1.8 h half-life, creating a possible radiation safety issue, particularly at higher beam intensities.

In some cases, dry nitrogen is used as an inexpensive sample environment gas with cooled samples to avoid condensation on sample windows. Nitrogen has only a slightly higher cross section with respect to air.

The cross section from gaseous water is considerably higher than air. But, increasing the humidity for air from a *T*_d_ = 283 K dew point to the much higher and nearly saturated humidity of *T*_d_ = 293 K only increases the air scattering cross section by 14%. The above comparisons were all computed at a λ_0_ = 10 Å neutron wavelength. The cross sections relative to each other have only a weak dependence on wavelength.

Calculating the background produced by gas scattering at the relatively small angles (θ ≤ 30°) used in SANS measurements requires more sophisticated modeling than that used for beam attenuation. Calculation of the distribution of angles and final energies after scattering can be done using more complicated expressions (Sachs & Teller, 1941 ▸). The measured scattering cross section, integrated over final energies, can then be approximated by the expression
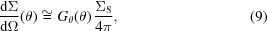
where *G*_θ_ is the normalized angular dependence of the gas scattering. As determined from TOF measurements in air, the final energy is not changed significantly from the initial energy, so the detector efficiency remains the same. The mean free path of neutrons in a gas varies from several to tens of metres, which is much larger than the lateral dimensions of the vessels. In this geometry, multiple scattering events are rare even for long path lengths. For liquid samples, further corrections are included in §3 to account for changes in detector efficiency for inelastic scattering and changes in *G*_θ_ caused by multiple scattering within the sample. The angular distribution is normalized such that
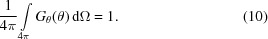



Now that we have calculated the expected corrections to the scattering cross sections, we return to measurements of the angle dependence of the absolute scattering cross section of gases. For gas measurements, the sample chamber was filled with He or Ar gas of 99.9% purity, filled with ambient air, or evacuated by vacuum pump. The data are put on an absolute scale by measuring the attenuated beam current *A*_f_∊_D_ *J*_B_ with the two-dimensional detector by removing the beamstop. The wavelength-dependent attenuation factor *A*_f_ is obtained using a lookup table derived from separate attenuator transmission measurements.

**Figure 2:**
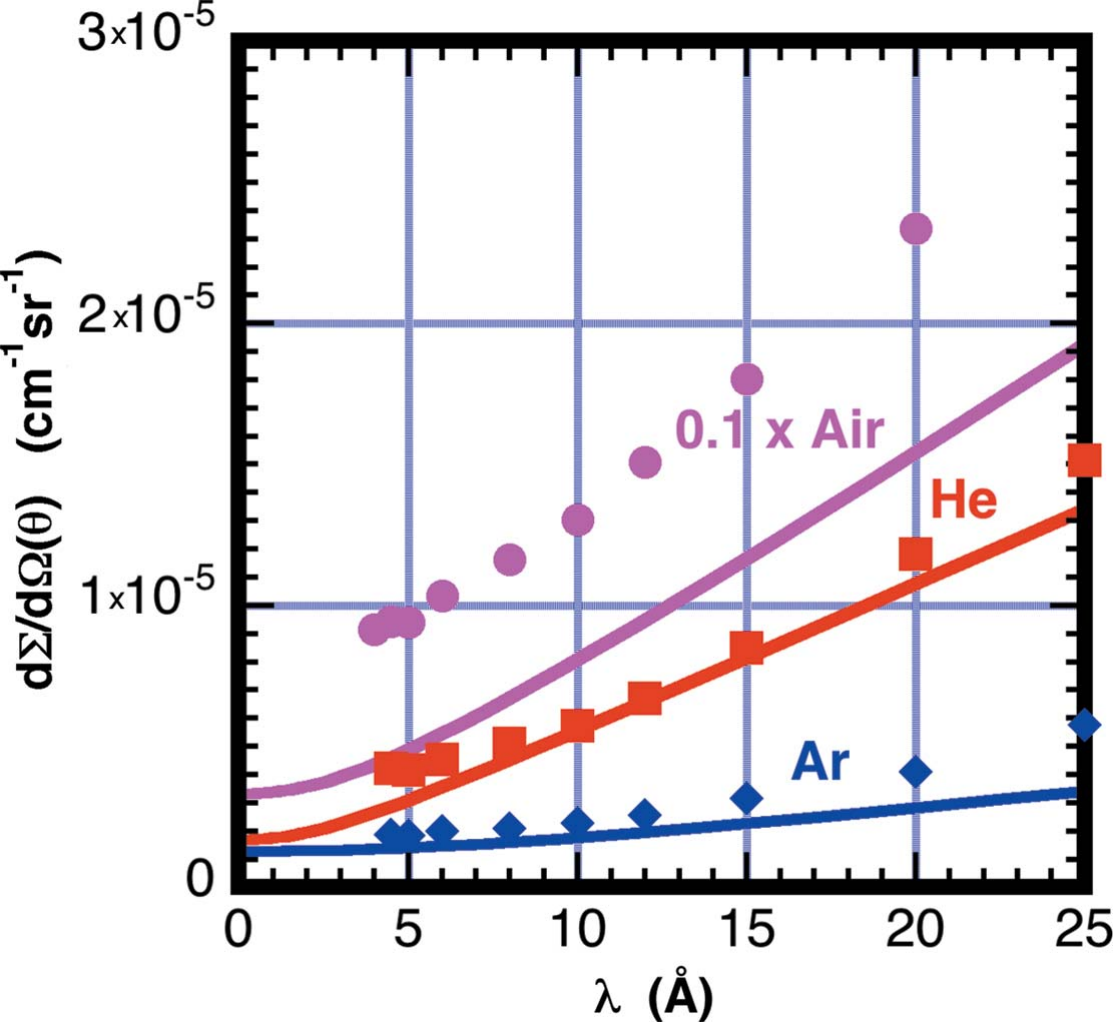
Measured scattering cross section from Ar (blue), He (red) and air (magenta) as a function of neutron wavelength. Measurements were made with an 18 cm path length of gas at room temperature and *P* = 1 × 10^5^ Pa and *L*_2_ = 1.33 m and by averaging the intensity on the detector over the scattering angle range 2 ≤ θ ≤ 24°. Solid lines are obtained using equations (6)–(9) [Disp-formula fd8] [Disp-formula fd9] with *G*_θ_(0) = 1. The air cross sections are rescaled by a factor of 0.1 to compress the vertical axis.

**Figure 3:**
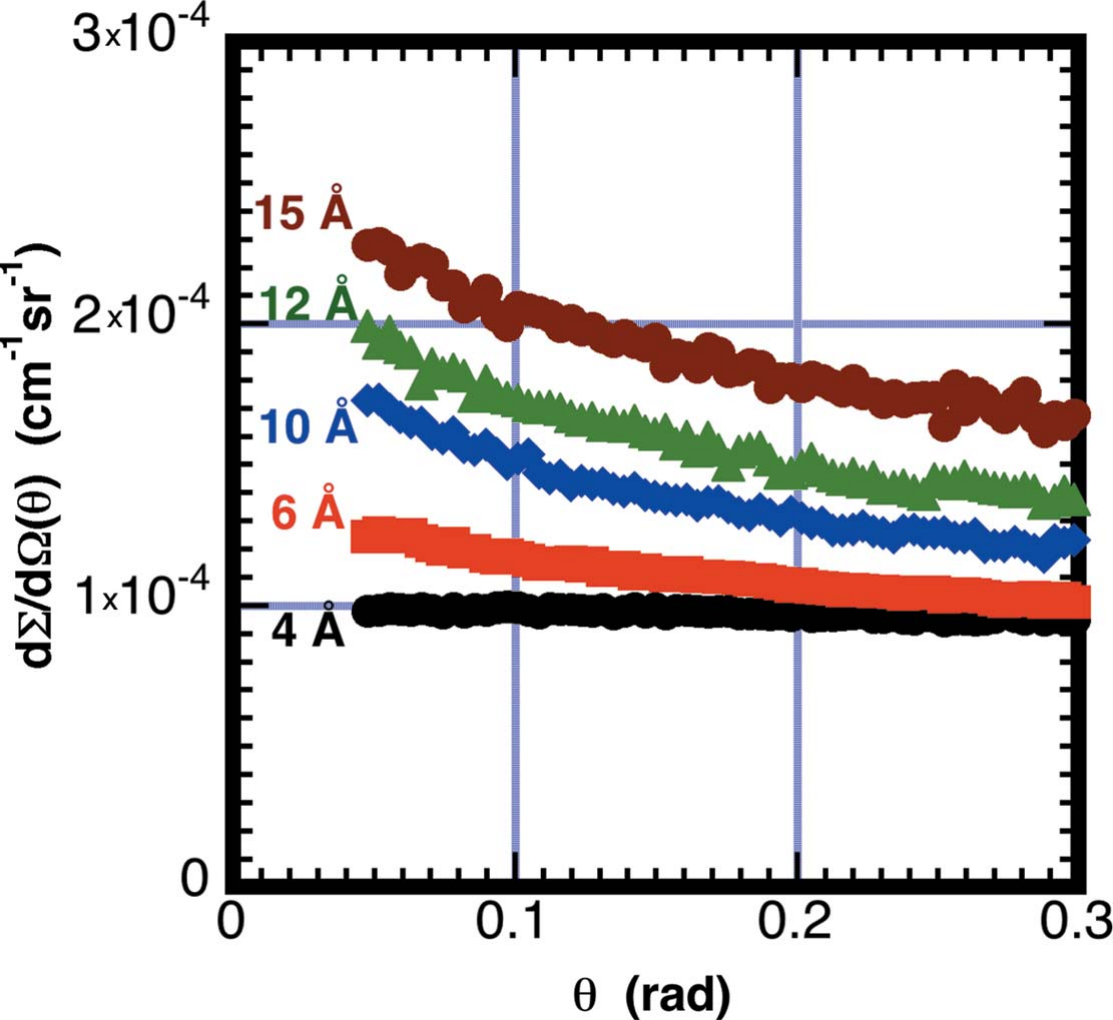
Measured scattering cross section from air as a function of neutron wavelength plotted *versus* scattering angle. Measurements were made with an 18 cm path length of air at room temperature and *P* = 1 × 10^5^ Pa and *L*_2_ = 1.33 m (symbols: λ_0_ = 4 Å black, 6 Å light red, 10 Å blue, 12 Å green and 15 Å dark red). Note that at longer wavelengths the scattering is observed to have stronger angular dependence.

The symbols in Fig. 2 ▸ correspond to the observed absolute scattering cross sections for Ar, He and air as a function of neutron wavelength, averaged over the entire detector with a scattering angle range 2 ≤ θ ≤ 24°, The lines are the cross section calculated using equation (9) and *G*_θ_ = 1. The observations are always higher than the calculation, indicating that *G*_θ_ > 1. The observed cross sections for air are considerably higher than the calculated solid curve by as much as a factor of two. Presumably this is due to forward scattering being enhanced, *G*_θ_(0) > 1. The enhanced scattering must be from the scattering cross section having higher probability in the forward direction. Fig. 3 ▸ shows air scattering as a function of scattering angle at various wavelengths. The scattering is found to decrease with scattering angle, particularly at longer wavelengths. This observed strong angular dependence for the scattering from air was not expected. Similar angle-dependent measurements from Ar and He do not show an angular dependence within the statistics of the measurement.

### 2.2. Evacuated vessel   

We now determine the expected effective scattering cross section for the residual air found inside the evacuated detector vessel. The enhancement in background comes from two factors: (1) the air scattering is integrated over the entire sample-to-detector path length, and (2) air scattering near the detector has enhanced solid angle coverage relative to near the sample position. Consider a beamstop of radius *R*_bs_ placed a distance *L*_bs_ in front of the detector. Air having pressure *P* is present over the entire distance *L*_2_ between sample and detector. At a detector pixel located a distance *R_i_* from the beam center on the detector, the beamstop shadows the pixel from scattering occurring at any distance *L* ≤ L* = *R_i_L*_bs_/(*R_i_* − *R*_bs_) from the detector. The cross section for the residual air must therefore be multiplied by a conversion factor *C*_F,*i*_ that can be obtained by integrating over the entire range of sample–detector distances:
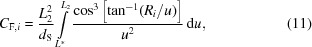
where cos^3^(θ*_i_*) accounts for the dependence of the scattering angle θ*_i_* on the solid angle subtended by a pixel on a flat detector, θ*_i_* = tan^−1^(*R_i_*/*u*), and (*L*_2_/*u*)^2^ accounts for the change in solid angle with distance from the detector.

**Figure 4:**
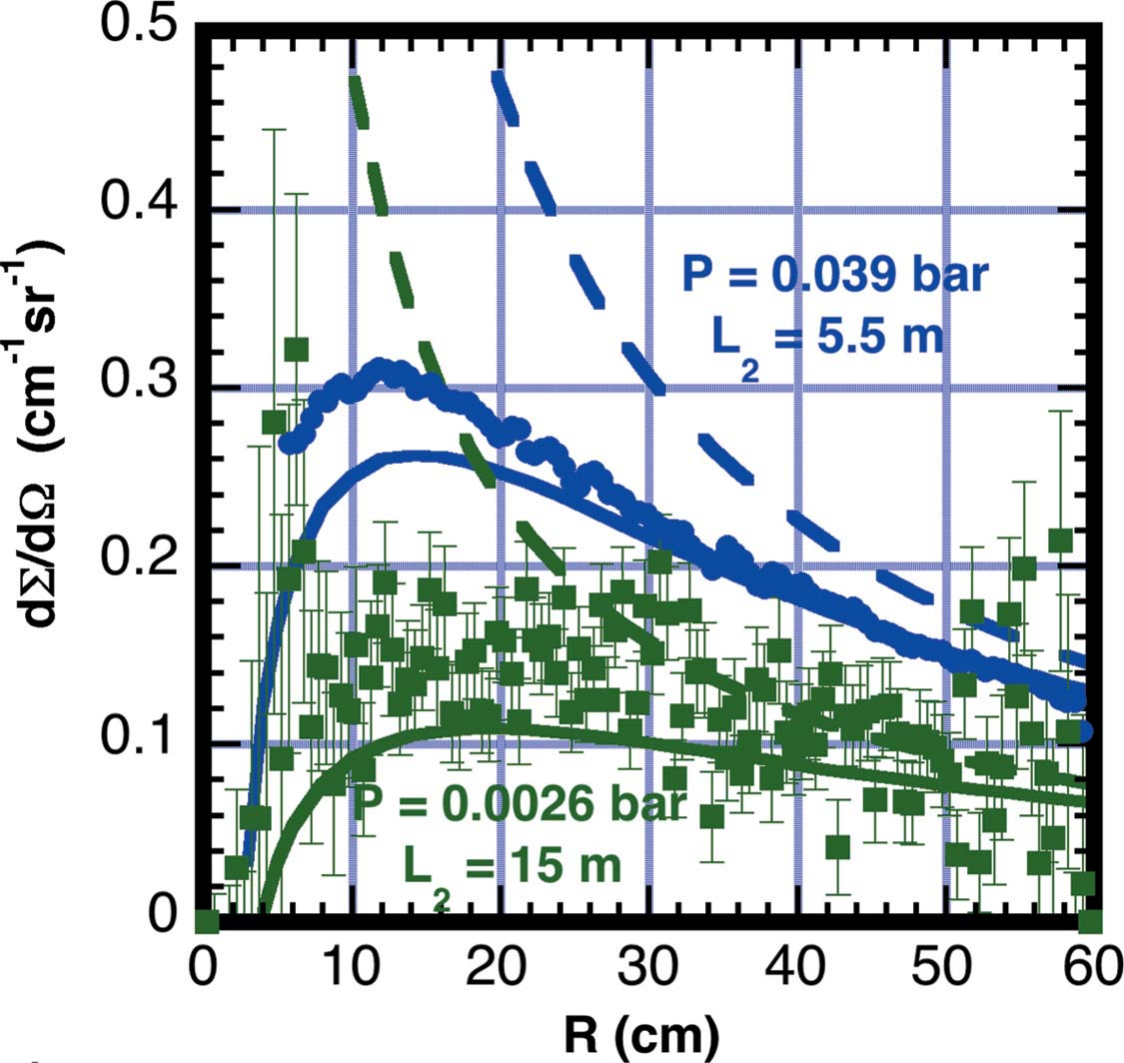
Measured effective scattering cross section from air in the detector vessel. Air with a pressure of *P* = 260 Pa was measured at a detector distance *L*_2_ = 15 m (green square symbols). An additional measurement was made with an air pressure of *P* = 3900 Pa and at a detector distance *L*_2_ = 5.5 m (blue circle symbols). Both data sets were reduced with a sample thickness *d*_S_ = 0.1 cm. The solid lines are calculated with a correction factor obtained from equation (11) with the beamstop located a distance *L*_bs_ = 26 cm in front of the detector. The dashed curves have the beamstop moved to the detection plane *L*_bs_ = 0 cm, thus removing the shadow of the beamstop.

To demonstrate the contribution of air scattering inside the detector vessel, the pressure inside the vessel was increased to produce detectable additional scattering. Background measurements were made on two separate occasions with a neutron wavelength λ_0_ = 6 Å and with the vessel evacuated to 2 Pa, and then with some air vented back into the vessel. During the first measurement, *P* = 3900 Pa air was added, with *L*_2_ = 5.5 m, *R*_bs_ = 2.5 cm and *L*_bs_ = 26 cm, and with the calculated maximum *C*_F,*i*_ = 8.2 × 10^4^ occurring at *R_i_* = 14 cm. Note that *P* = 3900 Pa of air scatters less than 1 × 10^5^ Pa of helium. For the second measurement *P* = 260 Pa air was added, with *L*_2_ = 15 m, *R*_bs_ = 3.8 cm and *L*_bs_ = 31 cm, and with the calculated maximum *C*_F,*i*_ = 5.1 × 10^5^ at *R* = 20 cm. Fig. 4 ▸ shows the measured effective backgrounds using a reference sample thickness of *d*_S_ = 0.1 cm. The measurements and calculations verify that for long flight paths residual air in a vacuum vessel can produce significant background scattering on the detector. We have found that maintaining a vacuum of *P* = 2 Pa shows benefits in reduced background for some experimental conditions. Backfilling with helium or argon is not recommended for the detector vessel.

### 2.3. Detector dome   

The ^3^He detector used in the measurements has a pressurized dome enclosing 2.4 × 10^5^ Pa ^4^He gas contained in a 20 cm-thick region in the direction of the beam. The front of the dome is made from a 0.48 cm-thick aluminium window. Neutrons can be scattered nearly isotropically either by phonons in the aluminium window or by the helium gas. The scattered neutrons can produce a ‘fogging’ effect in cases where strong scattering occurs near the beam direction. Some of this scattering can be rescattered by the dome, plus ^4^He gas, to be widely distributed over the detector, possibly obscuring weaker sample scattering. Normally the beamstop blocks the direct unscattered beam incident upon the dome, minimizing this effect. For some samples having a very large range in intensity from the near beam position to outer areas on the detector, this fogging effect has also been observed.

**Figure 5:**
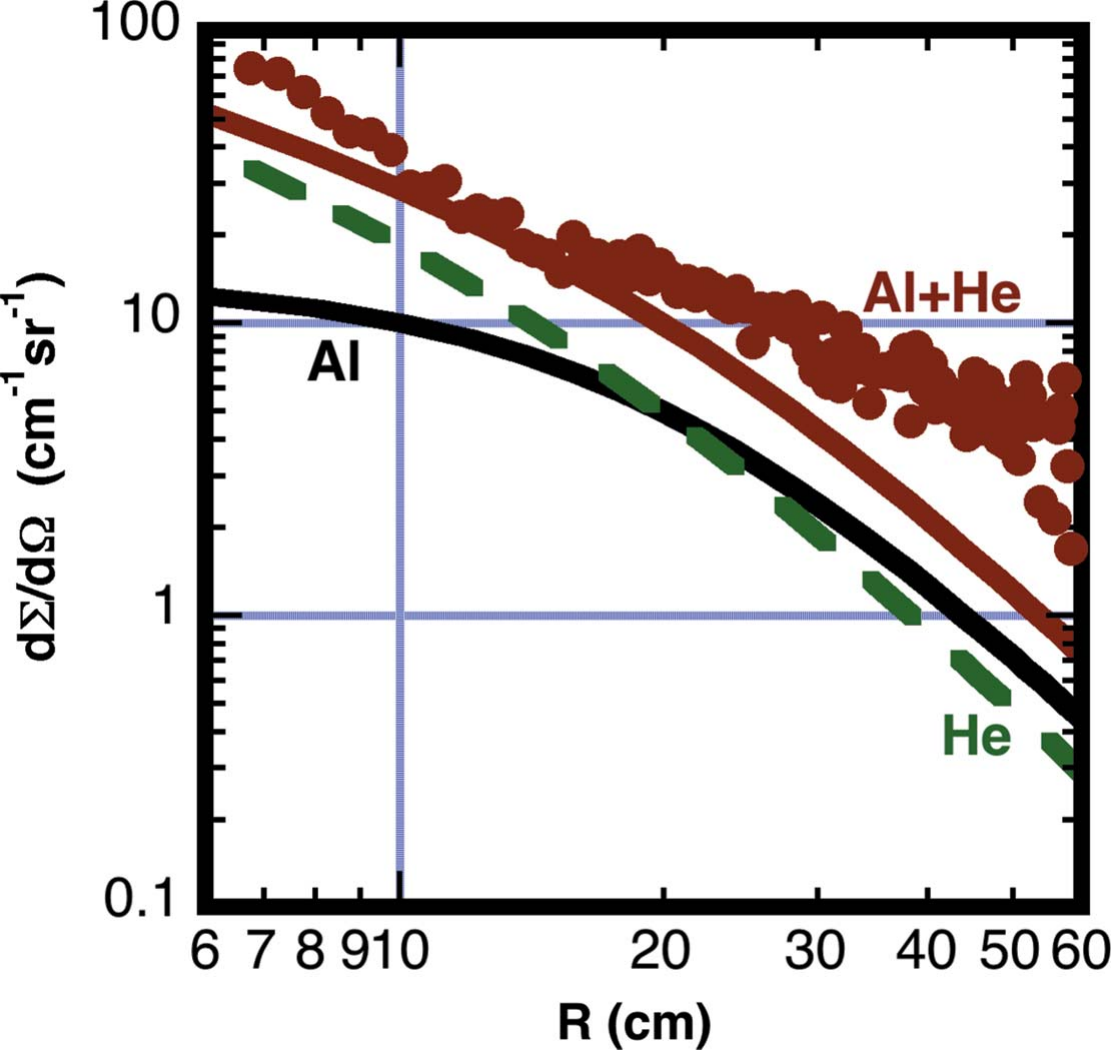
Measured effective scattering cross section from the dome of the detector, and calculated contributions from the ^4^He buffer volume and aluminium window. The scattering measurement was made with the attenuated empty beam and with the beamstop removed, allowing the beam to impinge on the detector dome.

To demonstrate the effect, measurements were made without a sample, but with the unscattered direct beam hitting the dome with the beamstop removed at a wavelength of λ_0_ = 6 Å. The neutrons scattered by the dome are easily seen as a scattered signal over the entire detector. The single-phonon scattering cross section from aluminium at room temperature is estimated from §4.3 of this paper as *I*(*q*) = Σ_sph_/4π = 5.2 × 10^−4^ cm^−1^ sr^−1^. The scattering from helium is obtained from Fig. 1 ▸ after rescaling for *P* = 2.4 × 10^5^ Pa to obtain *I*(*q*) = 1.1 × 10^−5^ cm^−1^ sr^−1^. The correction factor *C*_F,*i*_ is obtained using equation (11) and integrating from the front of the dome to the detection plane. Fig. 5 ▸ shows the observed parasitic background along with the calculated contributions from the aluminium and helium in the dome. Calculations were made assuming a sample thickness of *d*_S_ = 0.1 cm.

**Figure 6:**
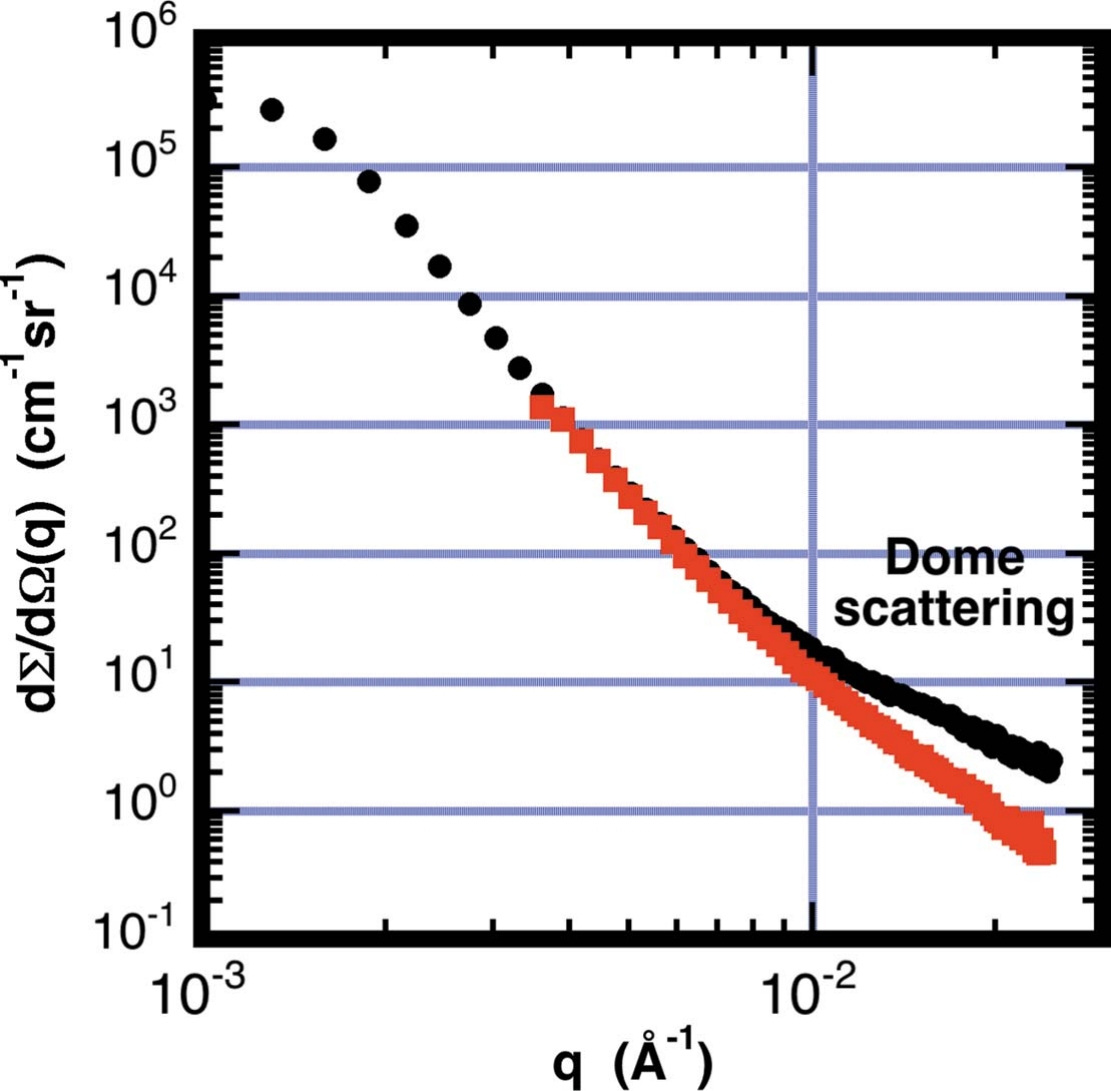
Scattering cross section from glass beads having a diameter of 35 µm. The measurements were made with two different size beamstops: 25 mm (black) and 100 mm (red). The larger beamstop partially shields the stronger scattering at small angles, lowering the detector count rate by 93%. The additional shielding also causes a reduction in the amount of dome scattering, evident in the mismatch between curves at large *q*.

To demonstrate dome scattering with a sample, 35 µm-diameter glass beads were measured. The refraction from the large particles produces Porod scattering *I*(*q*) ≃ *q* ^−4^. Two measurements were made: a 25 mm-diameter beamstop was used to block the direct unscattered beam, while a second measurement used a larger 100 mm-diameter beamstop to also block 93% of the scattered neutrons. Fig. 6 ▸ shows both measurements. Note that the 25 mm-beamstop data show a factor of five enhanced scattering at the edges of the detector. The additional scattering is not from the sample but is from neutrons scattered at smaller angles, within the shadow of the 100 mm-diameter beamstop, and then rescattered to the outer parts of the detector by the dome.

## 3. Scattering from liquids   

### 3.1. Theory and calculation   

SANS measurements are often made on solutions using light water (H_2_O), heavy water (D_2_O) or mixtures of the two. We present results for the total cross section determined from transmission and absolute scattering cross section *I*(*q*) measurements on the materials H_2_O, D_2_O (99.9% isotopic purity) and poly(methyl methacrylate) (PMMA) as a function of sample thickness and temperature. Detailed measurements and modeling of the neutron scattering from H_2_O and D_2_O are prolific in the literature since accurate data are needed for moderator design in reactors (see *e.g.* Mattes & Keinert, 2005 ▸). A number of models with good agreement with observed scattering exist for liquid scattering (Granada, 1985 ▸). The current measurements describe observations found in samples under typical conditions for SANS. The scattering from water samples as used in SANS measurements can also be simulated using sophisticated scattering kernels (Do *et al.*, 2014 ▸). TOF measurements, described in §4.3.3, were also made to determine the fraction of the scattering that is inelastic.

Scattering from 1 mm-thick samples of H_2_O is also often used as a secondary absolute scattering standard for SANS to put the scattered intensity on an absolute scale (Wignall & Bates, 1987 ▸). Several papers have identified that the measured cross section depends upon neutron wavelength (May *et al.*, 1982 ▸; Jacrot & Zaccai, 1981 ▸; Lindner, 2000 ▸) and sample thickness (Ghosh & Rennie, 1999 ▸; Shibayama *et al.*, 2005 ▸). Carsughi *et al.* (2000 ▸) have also demonstrated the dependence upon aperture and sample lateral dimensions by varying the beam and sample size with cadmium masks for both measurements and simulations.

The wavelength dependence of the detection efficiency will affect the measured scattering cross section since the scattering contains significant inelastic contributions. This effect has been demonstrated by Ghosh & Rennie (1999 ▸), who determined the inelastic spectrum from water and other materials using TOF. Since the typical detector has lower efficiency for inelastic scattered neutrons, we modify equation (9) to include a detector correction term,
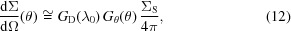
where *G*_D_(λ_0_) depends upon the specific detector efficiency response. The above expression is similar to that used to approximate the cross section from liquid water by Jacrot & Zaccai (1981 ▸), May *et al.* (1982 ▸) and Lindner (2000 ▸), where they calculate the combined parameter *G*_D_(λ_0_)*G*_θ_(0) from the measured absolute cross sections.

Since SANS data are typically put in absolute units using equation (2), where the scattered intensity is divided by the sample transmission *T*_S_, the observed cross section is amplified by a factor 1/*T*_S,S_ over that presented in equation (12) in cases where scattering is more likely than absorption. In such cases, *T*_S_ = *T*_S,S_ *T*_S,A_, where *T*_S,S_ and *T*_S,A_ are the sample transmission from scattering and absorption processes, respectively. Correcting (12) for the sample transmission used in (2) yields
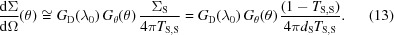
See Jacrot & Zaccai (1981 ▸) or Shibayama *et al.* (2005 ▸) for the derivation of the second relation in (13) in terms of sample transmission. Since the sample transmission *T*_S_ depends upon the sample thickness, equation (13) shows that the observed cross section as determined from standard SANS data analysis increases with sample thickness.

### 3.2. Multiple scattering   

SANS data are typically corrected for the scattering-angle dependence of the sample transmission *T*_S,θ_ caused by the change in path length. The angle-dependent transmission correction (Brûlet *et al.*, 2007 ▸) is
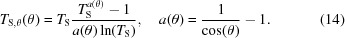
The above correction properly corrects the scattering cross section for single scattering events for slab sample geometry. However, the above transmission correction does not apply to multiply scattered events, which can be estimated by simply assuming the scattering is quasi-isotropic such that *G*_θ_(θ) = 1 for thin samples. Assuming that the sample can be approximated as an infinite slab, Chandrasekhar (1960 ▸) has developed a semi-analytical method to calculate the multiple scattering correction *G*_θ_ as a function of sample thickness *d*_S_, scattering Σ_S_ and absorption Σ_a_ cross sections.

To incorporate the sample geometry more accurately or to include a scattering kernel that has both energy and angular dependence, Monte Carlo simulations are usually necessary. Such simulations have been used to investigate the multiple scattering effects including absorption for typical water samples and for thin disc sample geometry. Carsughi *et al.* (2000 ▸) have investigated sample geometry effects thoroughly using both simulations and neutron measurements. These measurements show that sample thickness, beam size and sample size all affect the measured scattering cross section. From the current discussion, the specific sample geometry changes the contribution of multiple scattering, which then alters the function *G*_θ_(θ). Wider or taller samples multiply scatter a higher fraction of the neutrons in the forward direction, thus increasing *G*_θ_(0). Simulations that include the small absorption cross section for water and restricting the sample size to a disc do not significantly alter the results and are not included here. For example, an infinite slab water sample of 1 mm thickness, with λ_0_ = 5 Å, has a 0.6 (2)% higher simulated cross section *G*_θ_(0) than a sample having only 20 mm diameter, using a 15 mm-diameter beam. Sample geometry is expected to have a larger effect on *G*_θ_(0) with D_2_O owing to its longer neutron mean free path in comparison to its sample size.

**Figure 7:**
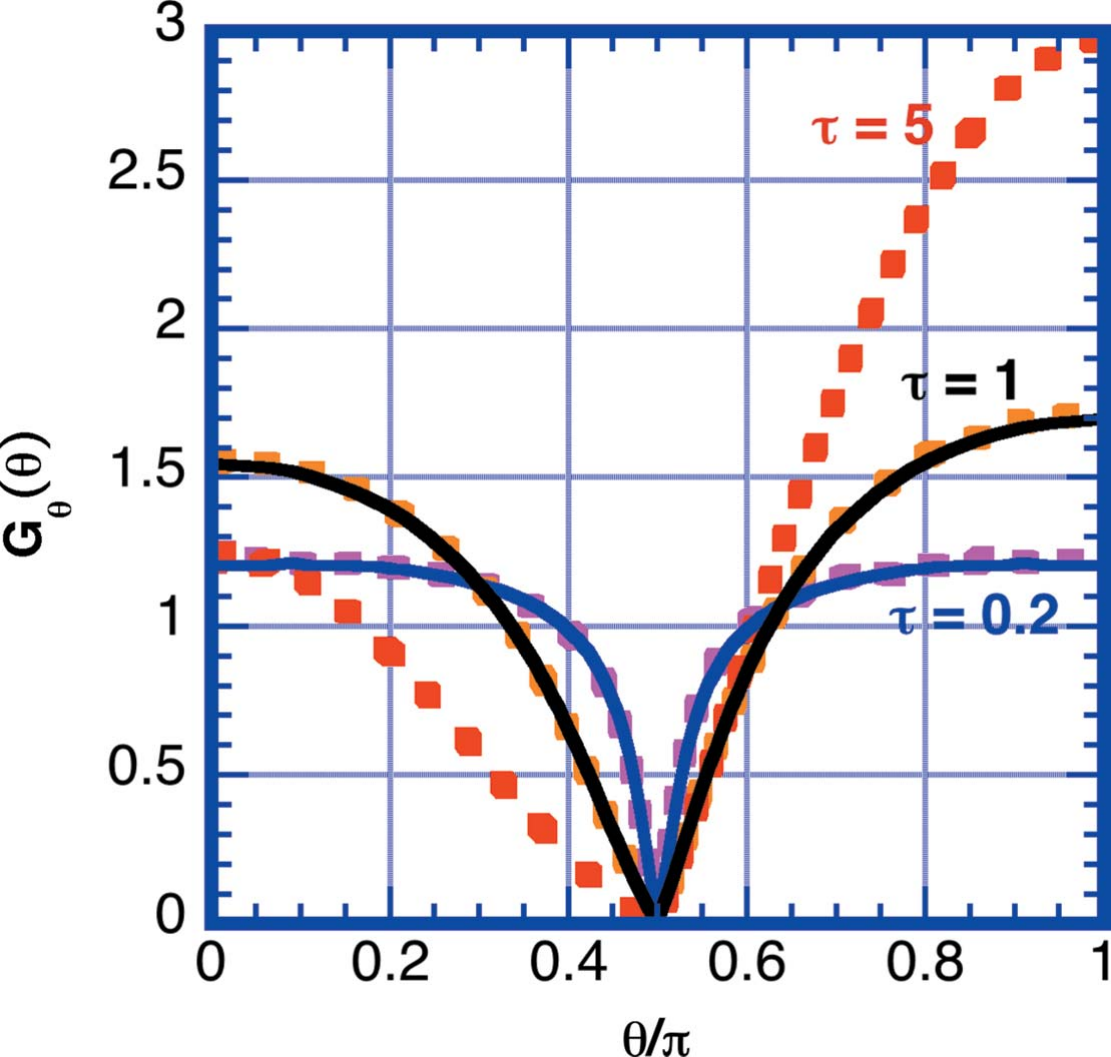
A plot of the multiple scattering correction factor for infinite slab sample geometry for an ideal sample exhibiting quasi-isotropic scattering without inelastic scattering or absorption. Data represented by dashed lines were obtained by simulation [τ = 0.2 (blue), 1 (black) and 5 (red)]. Data represented by lines were obtained using Chandrasekhar’s method and numerical data were obtained from Sobouti (1963 ▸).

**Figure 8:**
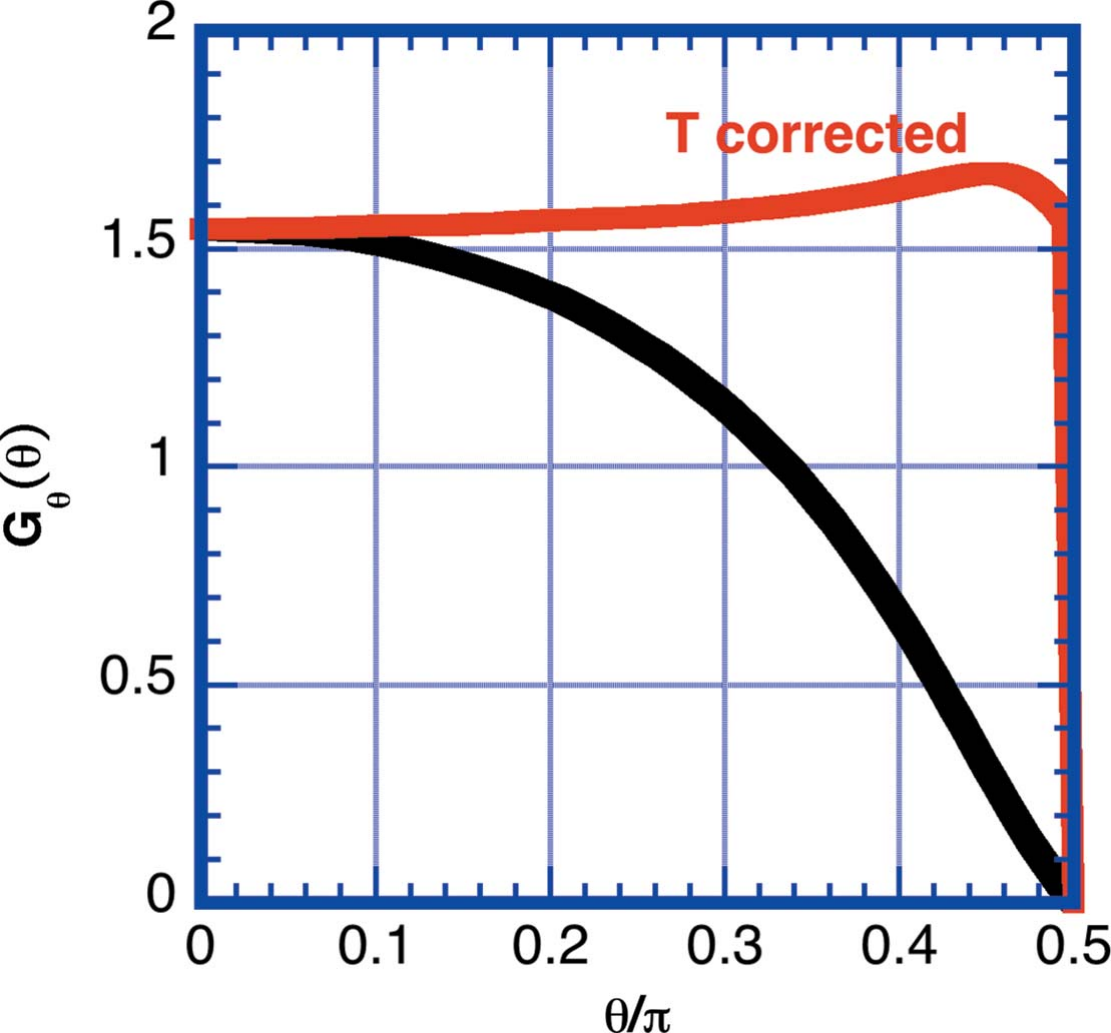
Data for τ = 1 replotted with angle-dependent transmission correction (14) applied (red) and without the correction applied (black). Note that the correction flattens the curve to a large extent, except at angles near 90° where the correction is large and multiple scattering is dominant.

The simulations presented here were conducted with no absorption, using a quasi-isotropic approximation assuming quasi-elastic scattering without inelastic scattering and in the infinite slab sample geometry to compare directly with the semi-analytical result of Chandrasekhar. Fig. 7 ▸ shows the simulated correction function *G*_θ_(θ) for scattering powers τ = Σ_S_ *d*_S_ = 0.2, 1 and 5. Over the wavelength range 5 ≤ λ_0_ ≤ 20 Å, at room temperature and sample thickness *d*_S_ = 0.1 cm, the scattering power for H_2_O varies as 0.5 ≤ τ ≤ 1.0 and for D_2_O as 0.06 ≤ τ ≤ 0.09. Note that higher scattering power preferentially pushes the scattering into the forward and backward scattering directions owing to the shorter path length through the sample in these directions. In Fig. 8 ▸ the transmission correction of equation (14) is applied to the simulated data. The correction does flatten the curve appreciably over the typical SANS measurement angle range 0 ≤ θ ≤ 30°. For example, at θ = 30°, the uncorrected curve has *G*(30°)/*G*(0°) = 0.93, but with the correction this ratio is 1.01. The error after correction increases as the sample thickness increases. For τ = 3 at θ = 30°, the deviation before correction is *G*(30°)/*G*(0°) = 0.84 and that after correction is 1.06. Note that the correction does not affect the intercept in the forward direction *G*_θ_(0).

**Figure 9:**
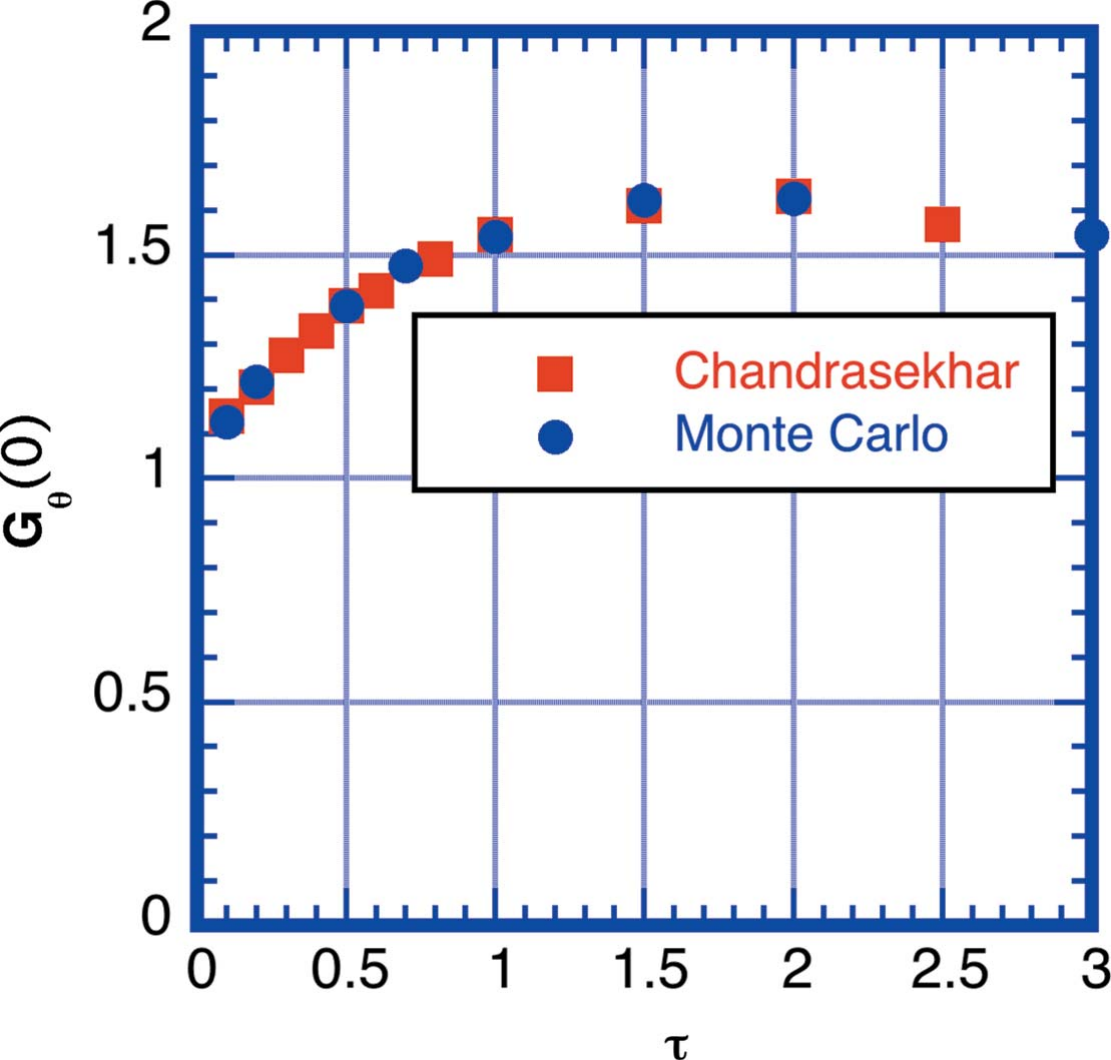
The multiple scattering correction factor in the forward scattering direction (θ = 0) for quasi-isotropic scattering as a function of scattering power τ. The data represented by circles were obtained by simulation and the data represented by squares by Chandrasekhar’s semi-analytical method. The sample geometry is an infinite slab. The scattering kernel assumes no absorption or inelastic scattering.

The amount of enhancement in the forward direction *G*_θ_(0) is also shown as a function of scattering power τ in Fig. 9 ▸. The enhancement reaches a maximum of 1.65 at τ ≃ 2 or *T* = exp(−2) = 0.135. The decrease at higher scattering power is due to self-shielding, which enhances the scattering in the backward direction. The calculation suggests that multiple scattering enhances the forward direction component by 50% for 1 mm thickness of H_2_O at λ_0_ = 6 Å. The simulations demonstrate that multiple scattering causes a scattering power or sample thickness dependence of *G*_θ_(0) and subsequently the measured scattering dΣ/dΩ(0).

### 3.3. Detector efficiency   

If all scattering were elastic, the detector efficiency correction would disappear, making *G*_D_ = 1 for all neutron wavelengths. But for liquids a large fraction of the scattering is inelastic. The detector efficiency correction is obtained by
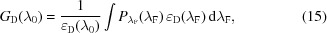
where λ_F_ is the scattered wavelength, ∊_D_ is the detector efficiency and *P*_λ_F__ is the probability distribution of scattered wavelengths λ_F_. The detector efficiency ∊_D_ for a typical SANS neutron detector can be approximated using the formula (Lindner, 2000 ▸):
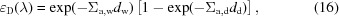
where Σ_a,w_ and Σ_a,d_ are the absorption cross section of the detector window material and the detection gas, respectively, and *d*_w_ and *d*_d_ are the thickness of the window and detection gas.

**Figure 10:**
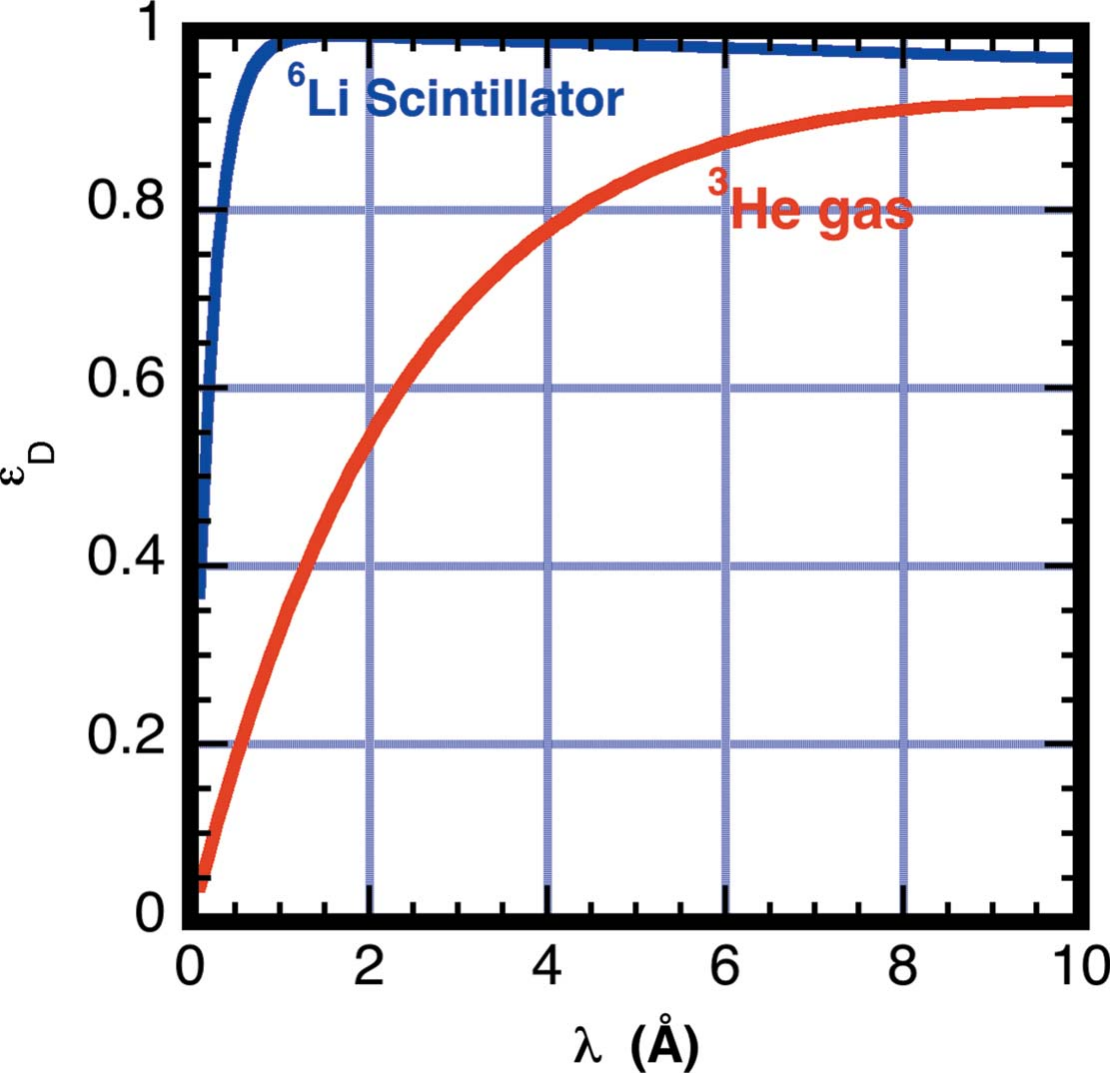
A plot of the calculated neutron detection efficiencies for the two different neutron detectors modeled in the text and with parameters given in Table 2 ▸ as a function of neutron wavelength λ_0_. The calculation is based on equation (16). Note that 2 mm of scintillator glass has much higher efficiency at thermal wavelengths than the ^3^He gas detector. The calculation probably overestimates the detection efficiency because discrimination of some valid events is likely owing to incomplete charge collection from the detector wall effect (Crane & Baker, 1991 ▸).

For comparison purposes, we calculate the *G*_D_ correction parameters for two dissimilar neutron detectors used for SANS measurements to highlight the large differences in detection efficiencies for inelastically scattered thermal neutrons. Note that the detection region thickness, composition and gas pressure affect the efficiency of the detector. The ^3^He detector is used at several facilities including the National Institute of Standards and Technology (NIST). The glass scintillator detector is in current use on the KWS2 instrument at Munich, Germany (Kemmerling *et al.*, 2004 ▸). Each detector design has a unique detector efficiency dependence on wavelength. Table 2 ▸ lists the values of the parameters in equation (16) used in these calculations. For both the ^3^He gas detector used in these measurements and the modeled ^6^Li scintillation detector, the window is composed of aluminium alloy having an absorption cross section Σ_a,w_/λ = 7.79 × 10^−3^ cm^−1^ Å^−1^. The detection gas pressure for the ^3^He detector is 2 × 10^5^ Pa. Eighteen atomic percent ^6^Li_2_O in an SiO_2_ matrix having 2.5 g cm^−3^ mass density is used for calculating the scintillator glass detector efficiency. Fig. 10 ▸ shows the calculated detector efficiencies for the two detectors.

**Figure 11:**
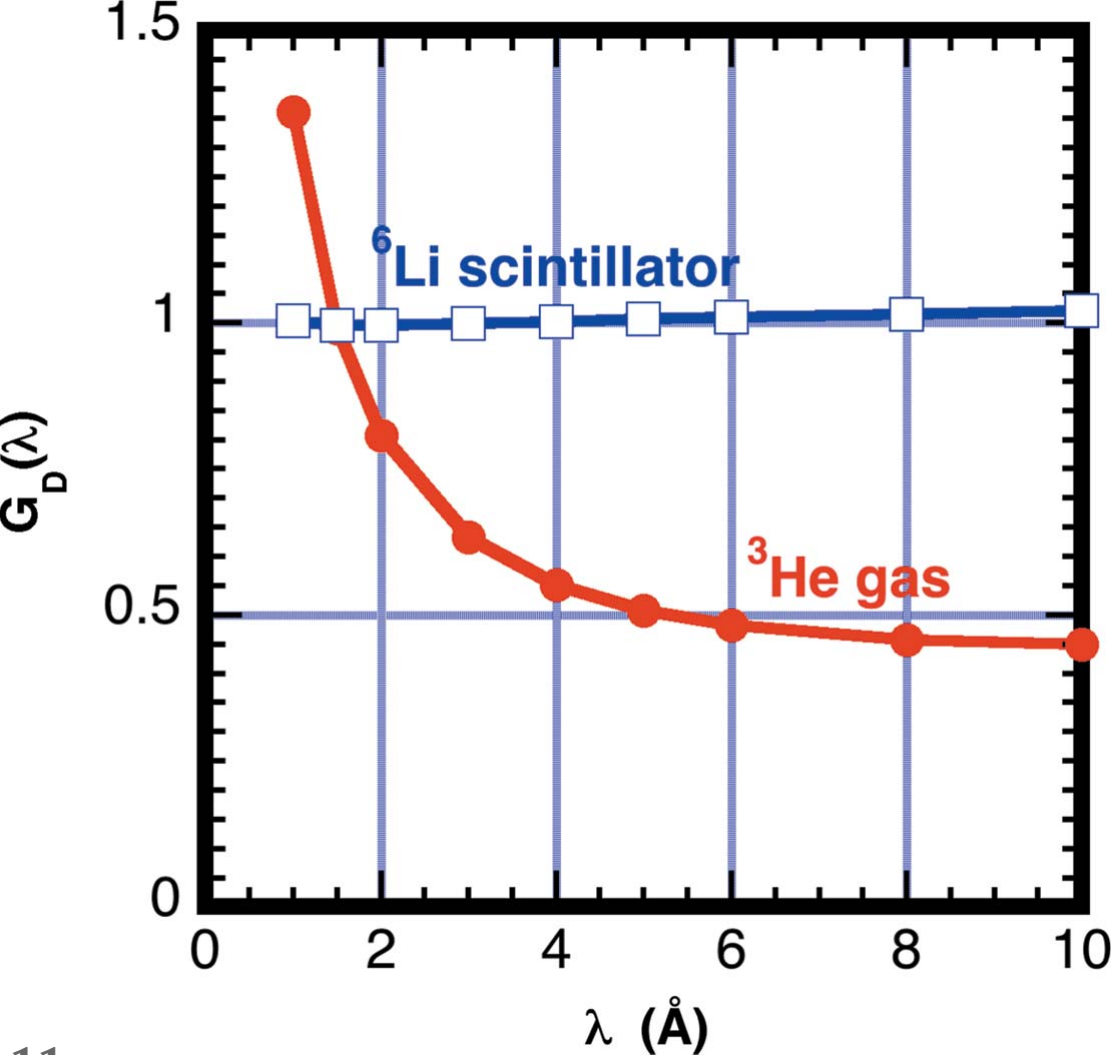
A plot of the detection efficiency correction parameter assuming all scattering is inelastic (*f*_in_ = 1) and is distributed in energy as a Maxwellian having temperature *T* = 300 K. The calculation was performed for each of the two different detectors. The appropriate correction for a smaller fraction of inelastic scattering (*f*_in_ < 1) is *f*_in_ *G*_D_ + (1 − *f*_in_).

The *G*_D_ correction depends upon the energy dependence of the scattering. We greatly simplify the calculation by assuming that the scattering can be separated into two components: quasi-elastic events, where the initial and final energies are nearly the same, and inelastic events, comprising the rest of the scattering. The fraction of all scattering events that is inelastic is *f*_in_. Assuming a simple Maxwellian distribution in final neutron energy or wavelength for inelastic events,
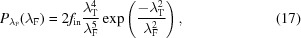
where λ_T_^2^ = 953 Å^2^ K/*T* and *T* is the temperature of the distribution. The remaining events have 1 − *f*_in_ probability caused by quasi-elastic scattering having λ_F_ = λ_0_. At room temperature, *T* = 293 K, the characteristic wavelength λ_T_ = 1.80 Å. This distribution approximation for inelastic scattering improves for thicker samples, where multiple inelastic scattering tends to change the shape of the energy dependence of the scattering spectrum to more closely resemble a Maxwellian distribution. (TOF experimental observations discussed later approximate a simple Maxwellian distribution.) From the distribution of neutron energies on the detector obtained from equation (17), and the detection efficiency at each energy obtained from equation (16), we can determine the correction to the mean detection efficiency in *G*_D_(λ_0_) using equation (15). Fig. 11 ▸ shows the correction factor *G*_D_ for each of the two different detectors as a function of incident neutron wavelength λ_0_, assuming that all scattering is inelastic with a room-temperature Maxwellian distribution. Note that the correction at long wavelengths becomes sizable for the ^3^He detector, which has lower efficiency at short wavelengths. If 50% of the neutron events are inelastically (*f*_in_ = 0.5) scattered at a neutron wavelength λ_0_ = 6 Å, then for the helium detector *G*_D_ = 0.5 × 0.48 + (1 − 0.5) × 1.0 = 0.74. For the scintillation detector the estimate has *G*_D_ = 1.00. The cross section from 1 mm-thick water samples measured with a scintillation detector is expected to be a factor 1.0/0.74 = 1.35 higher than that measured with the helium detector.

**Figure 12:**
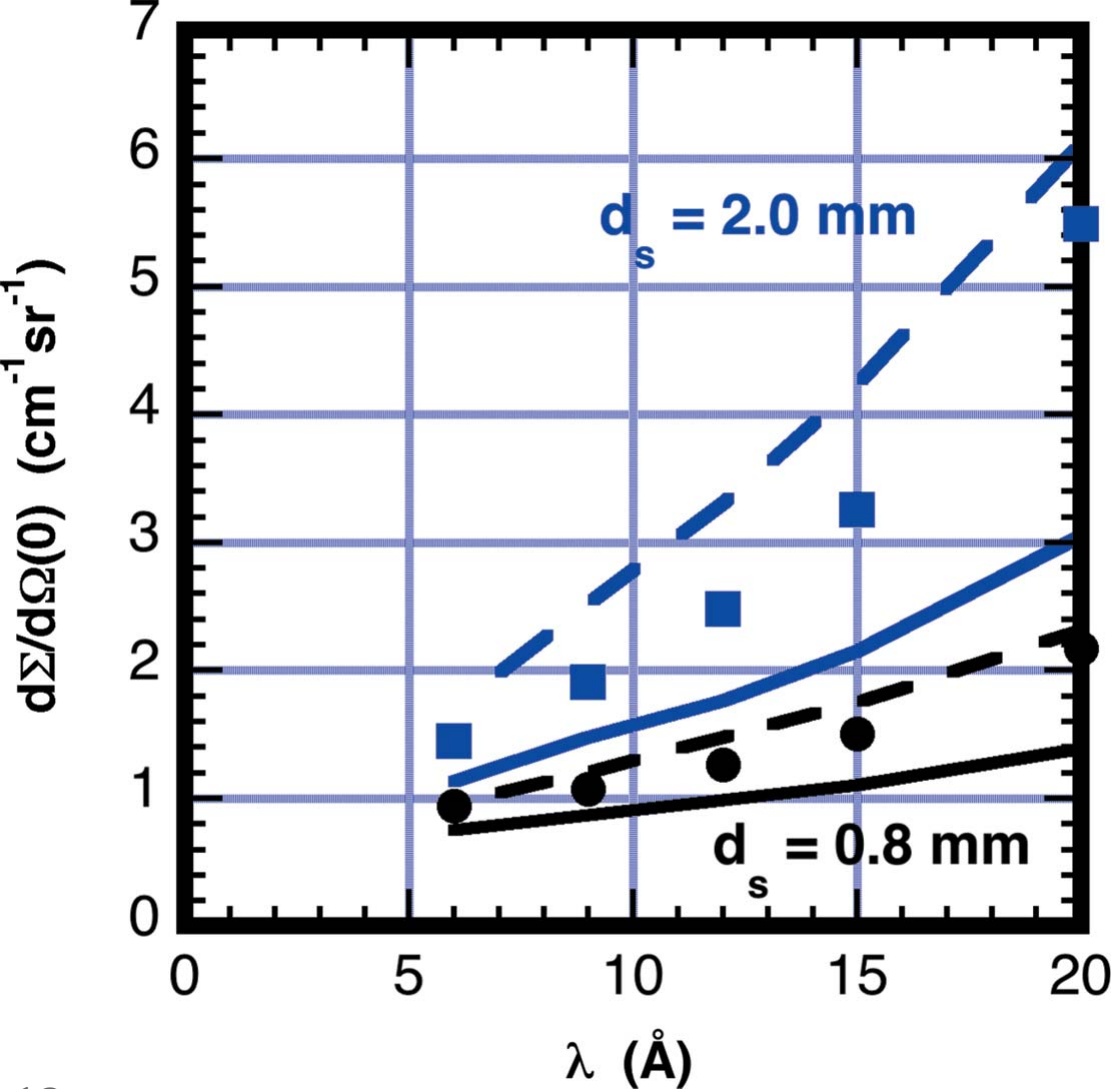
Thickness and wavelength dependence of the forward scattering from water as averaged over the whole ^3^He 0.64 × 0.64 m area detector with *d*_S_ = 0.8 mm (circles) and 2.0 mm (squares). Data are put on an absolute scale using equation (2) and the beam current *J*_B_ as measured using a ^3^He detector with calibrated beam attenuation. The solid lines are calculated according to equation (13), with *G*_D_ for the helium detector as obtained from Fig. 11 ▸ and *G*_θ_ obtained from Fig. 9 ▸. Black and blue denote 0.8 and 2.0 mm thicknesses, respectively. The scattering power τ is obtained from the sample transmission and inelastic fraction obtained from TOF using Table 6 ▸. The dashed curves use the scintillation detector efficiency to calculate G_D_. The increase in scattering observed with 2 mm thickness is primarily a result of the lower transmission.

Fig. 12 ▸ plots the measured scattering from 0.8 and 2.0 mm-thick deionized H_2_O samples measured over the wavelength range of 5–20 Å. The curves on the plot show the cross sections obtained using equation (13). The values for *G*_D_ *G*_θ_ were obtained using Figs. 9 ▸ and 11 ▸. The calculation underestimates the measured values by 20–40%.

The total scattering cross sections for 0.8 mm-thick H_2_O and 4 mm-thick D_2_O were measured over the wavelength range of 5 ≤ λ_0_ ≤ 20 Å with sample temperatures of *T* = 298, 323, 348 and 368 K. The measured scattering cross section increases by 40% at λ_0_ = 20 Å when the temperature is increased from 298 to 368 K. Note that the density of H_2_O decreases by 3.5% over this temperature range, whereas the increase at λ_0_ = 5 Å is negligible. The scattering increase at the higher temperatures comes from inelastic processes, so that the background at long wavelengths depends on temperature. Consequently shorter wavelengths are preferred to minimize the background.

### 3.4. Deuterated samples   

Coherent scattering from mixtures of deuterated and hydrogenated solvents is described by Arleth & Pedersen (2000 ▸). Deuterium has a smaller incoherent cross section (σ_inc_ = 2.0 b; 1 b = 10^−28^ m^2^) than hydrogen (σ_inc_ = 79.7 b); consequently D_2_O has a smaller total cross section Σ_T_ and scattering cross section *I*(*q*). Samples can be up to ten times thicker because of the lower attenuation. Background scattering is also lower by a factor of up to 20 for samples having a small volume fraction of solute. To obtain strong contrast the solute is often hydrogenated.

A significant fraction of the observed scattering from D_2_O is from coherent processes. The liquid structure factor produces a broad scattering peak centered at *q* = 2.0 Å^−1^ (Thiessen & Narten, 1982 ▸). At wavelengths λ_0_ > 6 Å, the *q* range excludes scattering from the peak, which both reduces the beam attenuation and reduces forward scattering produced by double scattering events. The total cross section Σ_T_ from D_2_O has a local minimum at λ_0_ = 9 Å due to the exclusion of the scattering peak. A similar effect is seen for quartz, which has a coherent peak from its glass structure near *q* = 1.55 Å^−1^ (Root *et al.*, 1990 ▸).

The coherent cross section also produces forward scattering *I*_f_(0) in liquids and glasses from thermal fluctuations. The absolute contribution from the fluctuations depends upon the material compressibility χ_T_ as (Orthaber *et al.*, 2000 ▸)
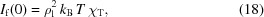
where ρ_l_ is the scattering length density of the material. Forward scattering *I*(0) from fluctuations in liquids is used as an absolute standard in small-angle X-ray scattering (SAXS) measurements since inelastic and incoherent cross-section contributions are negligible in comparison. Table 3 ▸ provides both SANS and SAXS forward cross sections for H_2_O, D_2_O and quartz. For neutrons, only D_2_O has an appreciable (∼10%) contribution to the forward scattering from fluctuations.

## 4. Scattering from crystalline solids   

### 4.1. Double Bragg scattering   

Strong background scattering at small angles in X-ray or neutron scattering measurements can also be produced by Bragg diffraction from a crystalline reflection in one part of the sample, followed by a second Bragg diffraction of negative order to ‘back reflect’ in a different part of the sample. If both regions are part of the same crystal with no misorientation, also known as a perfect crystal, the radiation direction returns to the original unscattered ray direction and is indistinguishable from the unscattered beam; this is sometimes called ‘null’ scattering. However, if the two regions are part of the same crystal but have a small misorientation with respect to each other, the second scattering causes the ray direction to also have a small misorientation from the original unscattered ray. The misorientation in different regions of a crystal can be caused by mechanical strain or by other defects in the crystal such as strain from coherent precipitates, small-angle tilt boundaries or other dislocation structures. Double Bragg scattering (DBS) from a single crystal can manifest itself as streaks radiating out from the beamstop, as observed in SAXS by Buckley *et al.* (2001 ▸). In cases where more than one single-crystal window of nominally the same orientation are stacked together, one or more Bragg spots outside the beamstop have also been observed.

In polycrystalline solids that do not have a preferred texture, the DBS appears azimuthally symmetric with power-law *q* dependence *I*(*q*) ≃ *q* ^−*m*^. Warren (1960 ▸) has shown theoretically that *m* = 1 for randomly oriented grains, whereas Taglauer (1968 ▸) observed *m* > 1 from SANS observations of polycrystalline copper. Presumably the higher cross section and *q* dependence are due to the orientation distribution of the crystalline grains not being random.

A reliable method for determining the presence of DBS in any sample is to make measurements at different wavelengths or single-crystal orientations, both at the Bragg scattering condition and where Bragg scattering is not possible. The difference in the observed scattering at small angles can thus be attributed to DBS. For neutron measurements with cold neutrons, the simplest method to eliminate Bragg scattering is to choose the wavelength λ_0_ > 2*d*_max_, where *d*_max_ is the maximum *d* spacing for allowed reflections in the crystal structure.

**Figure 13:**
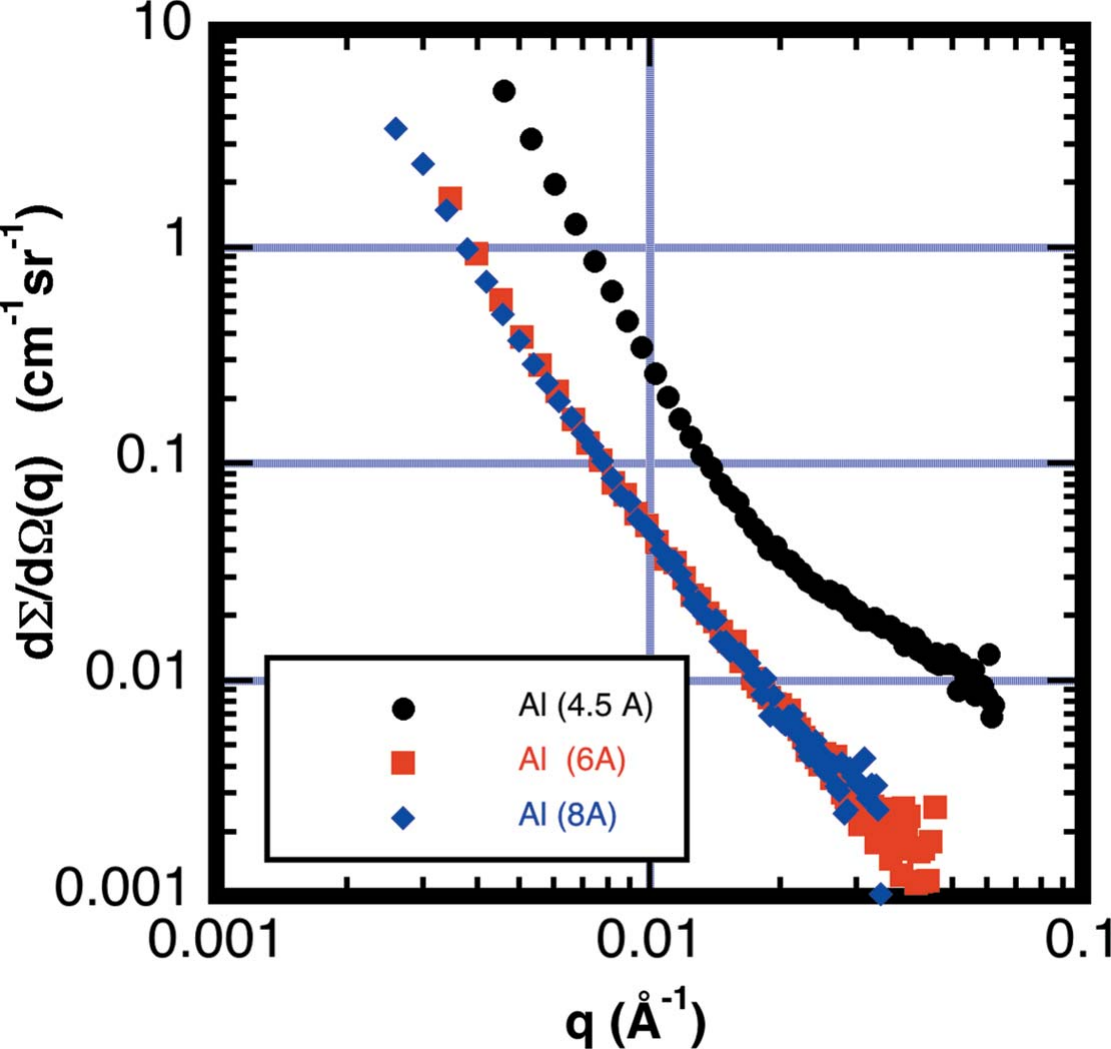
Measured cross section from 30 sheets of 1 mm-thick 99.99% pure polycrystalline aluminium sheet at wavelengths λ_0_ = 4.5, 6 and 8 Å, with wavelength spread Δλ/λ = 22%. Double Bragg diffraction is observed only at λ_0_ = 4.5 Å, where the Bragg condition is active for the *hkl* = 111 reflections in the face-centered cubic crystal structure. The scattering at longer wavelengths is likely to be due to the dislocation structure.

Fig. 13 ▸ shows the scattering from a 99.99% pure aluminium polycrystalline sheet (30 layers of 1 mm-thickness sheet stacked together) taken at three different neutron wavelengths, λ_0_ = 4.5, 6 and 8 Å, each with wavelength spread Δλ/λ = 22%. For aluminium, *d*_max_ = 2.338 Å, so Bragg scattering occurs for wavelengths λ_0_ < 2*d*_max_ = 4.676 Å. The observed scattering curves at λ_0_ = 6 and 8 Å overlap with each other, while the data taken at λ_0_ = 4.5 Å have much higher intensities caused by DBS. Similar scattering curves are also observed from a 99.99% pure niobium polycrystalline sheet, while scattering from a sapphire single crystal produces only a strong Bragg spot at λ_0_ = 4.5 Å. The spot disappears at the λ_0_ = 8 Å wavelength. All three samples exhibit strong additional scattering at λ_0_ = 4.5 Å caused by DBS when compared to the longer wavelengths.

The crystal orientation with respect to the beam for single-crystal windows or samples can also be chosen to eliminate Bragg scattering. For example, silicon wafers can be obtained commercially with a crystal orientation with respect to the surface normal of (111), (110) or (100). The longest wavelengths that diffract from each orientation are λ_0_ = 6.271 Å for (111), λ_0_ = 5.120 Å for (110) and λ_0_ = 3.621 Å for (100). Silicon windows having (100) orientation are preferred since shorter wavelengths can safely be used.

### 4.2. Dislocations and precipitates   

The remaining scattering at the longer wavelengths in Fig. 13 ▸ is probably dominated by scattering from the dislocation structures as predicted by calculations made by Schmatz *et al.* (1974 ▸) and seen in more recent experimental results on copper by Heuser (1994 ▸). Scattering from isolated edge dislocations of random orientation should follow the power law *I*(*q*) ≃ *q* ^−3^. Grain boundaries typically produce very weak scattering since the atomic density of the grain boundary region is nearly the same as in the interior of the grain; however, this background scattering can be enhanced by solute segregation or magnetic demagnetization by the strain field. Solute atoms can be attracted to the strain field in or near the dislocation core, thus causing enhanced nuclear contrast, as seen in hydrogen charging of palladium (Maxelon *et al.*, 2001 ▸), for example. The strain from the dislocation core causes demagnetization of ferromagnetic materials near the dislocation core, creating strong magnetic scattering contrast (Gerold & Kostorz, 1978 ▸). A stronger source of background in ferromagnetic materials is the refraction from magnetic domain walls. SANS measurements on ferromagnetic alloys such as steels or nickel-based superalloys should be performed in a strong magnetic field to saturate the magnetization of the sample where the background scattering can be reduced by two or more orders of magnitude.

Background scattering from precipitates or voids in solid materials is a common problem. Oxygen precipitation in otherwise high-purity silicon single crystals grown by the Czochralski (Cz) method is known to occur, particularly under high-temperature annealing conditions (Borghesi *et al.*, 1995 ▸). A Cz-grown silicon single-crystal window was removed from service at NIST after the discovery of scattering that fitted the profile expected from small plate-like precipitates. Commercially pure materials often produce strong scattering from precipitates formed from impurities. For metallic alloys, the precipitates often provide superior mechanical strength over a higher-purity material. Thus sample environments on neutron instruments that measure inelastic scattering often use precipitation-strengthened aluminium alloys such as 6061-T6, which produce copious background on SANS instruments. Materials such as titanium grade 5 containing a mass fraction of 6% V and 4% Al, which are strengthened by atomic substitution, fine grain size and large dislocation density, can provide the higher yield strengths needed with smaller additions to the background as compared to precipitation-strengthened alloys. In addition, our experience has been that degassing of solvents has significantly reduced background from bubbles too small to be seen by eye in liquid samples. Inclusions and voids in low-quality glasses can also produce enhanced background scattering (see Porcar *et al.*, 2011 ▸). Optical quality windows made from quartz typically show smaller amounts of scattering.

Additional scattering may come from the sample surface (Roth, 1977 ▸). Background from surfaces can be sizeable if many samples are stacked together or if the surface has excessive roughness. Polishing the surface to an optical finish usually reduces the background to an undetectable level. Such polishing can enhance DBS if residual stresses are produced. Cracks in the sample can either reflect or refract the beam, usually evident as asymmetrical streaks on the detector.

### 4.3. Inelastic scattering   

#### 4.3.1. Theory and calculation   

Methodology similar to that used here in developing the single-phonon cross sections is also described by Freund (1983 ▸) and Barker *et al.* (2008 ▸). Neglecting incoherent and Bragg scattering, the total macroscopic cross section Σ_T_ can be separated into three attenuation processes: (1) temperature-independent absorption by the nuclei, (2) single-phonon scattering and (3) multiple-phonon scattering:
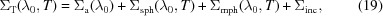
where Σ_inc_ is the incoherent cross section. The absorption cross section is related to wavelength by
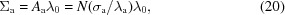
where *N* is the number of molecules per unit volume of material and σ_a_ is the thermal absorption cross section per molecule at the standard tabulation wavelength λ_0_ = 1.798 Å.

Steyerl (1977 ▸) has given an expression for the calculation of the temperature-dependent single-phonon scattering as a function of temperature *T* that depends on the Debye temperature Θ_D_ and the total scattering cross sections of the constituent atoms. This expression has been used for neutron filter materials such as Al_2_O_3_ (Mildner & Lamaze, 1998 ▸), MgO (Thiyagarajan *et al.*, 1998 ▸) and MgF_2_ (Barker *et al.*, 2008 ▸).

The Debye temperature for a given material is often found to differ somewhat depending upon the specific measurement technique and temperature. Table 4 ▸ gives the values of Θ_D_ for the materials obtained from Ahlers (1966 ▸) for Be, Striefler & Barsch (1975 ▸) for quartz and Batterman & Chipman (1962 ▸) for Si, whereas values for sapphire and MgO are those used by Mildner & Lamaze (1998 ▸) and Thiyagarajan *et al.* (1998 ▸), and those for Al, Cu, Ge, Mg and Nb are from Alers (1965 ▸).

Another contribution to the inelastic cross section is an interference term to the phonon contribution, as described by Binder (1970 ▸), which at long wavelengths is proportional to wavelength and can be negative or positive. Binder’s calculations show a negative contribution for bismuth and a positive contribution for aluminium. For aluminium, the interference term is 15% of the phonon contribution. To both include this interference term and accommodate possible errors in the Debye temperatures used, the total cross sections determined from the transmission measurements as a function of temperature are fitted to a modified expression:
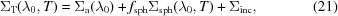
where Σ_a_(λ) and Σ_sph_(λ_0_, *T*) are functions defined by fixed parameters calculated simply from material constants, and only *f*_sph_ is allowed to change to account for possible interference contributions to the cross section. Note that an error in the Debye temperature can also systematically shift the fitting parameter *f*_sph_ up or down. Barker *et al.* (2008 ▸), using the literature value for the Debye temperature Θ_D_ = 610 K, found that the fit produces *f*_sph_ = 1.20 (5), whereas if the Debye temperature in the fit is shifted to Θ_D_ = 520 K, the data could be fitted with *f*_sph_ = 1.

The multiphonon scattering that dominates at shorter wavelengths has been both modeled theoretically by Freund (1983 ▸) and fitted empirically by others. Here, we concentrate our modeling on cold neutrons, λ_0_ > 5 Å, where the multiphonon scattering cross sections have a small contribution; thus, the multiphonon contribution has been omitted from equation (21).

#### 4.3.2. Measurement   

We have performed neutron transmission measurements on the various materials listed in Table 4 ▸ as a function of temperature from *T* = 293 to 750 K, and with neutron wavelengths λ_0_ = 10 and 20 Å. The scattering cross section was also measured at λ_0_ = 10 Å with a detector distance *L*_2_ = 2 m. The samples were loaded in a multiple-position sample holder covered in 20 layers of aluminium foil to reduce heat loss and reduce temperature gradients. The sample chamber was evacuated to further reduce temperature gradients from air convection. Measurements were made in 50 K temperature increments. Two different sample holders were used. The majority of measurements were made with a seven-position aluminium sample holder having a maximum temperature *T* = 550 K. Higher-temperature measurements up to 750 K were made using a two-position titanium sample changer on all samples in Table 4 ▸ except for Ge. The polycrystalline samples were Al, Be, Cu and Nb. The single-crystal samples were sapphire, C (graphite), Ge, MgO, MgF_2_ and Si. The crystal orientation with respect to the beam was the *c* axis for sapphire and graphite, (220) for Ge and Si, and unknown for MgO and MgF_2_. All the crystals have high perfection except for the C (graphite) sample, which has a mosaic spread (FWHM) of 2°. The quartz windows were of optical quality.

The quality of the fit of Σ_T_ to equation (21) for any given material varies if the material exhibits strong absorption, because it is difficult to observe the smaller relative change in the phonon cross section with temperature. If the sample exhibited strong SANS, the transmission was affected by the *q* range over which the transmission was integrated. Transmission data were collected with the area detector at both 4 and 15 m from the sample. Transmissions were determined by summing both over the area of the direct beam and over the entire detector. All transmission data used in fits were collected with the detector at the 4 m distance and summed over the entire detector, to collect nearly all the SANS events. The standard deviation in the cross sections σ_Σ_ was determined by using shot noise statistics and propagating the error contributions from both sample and empty measurements. The relative error, the standard deviation divided by the total cross section σ_Σ_/Σ_T_, is less than 2%, and it is less than 0.5% for thicker samples or higher temperatures where the transmission was lower. The uncertainty in determining the fitted parameter *f*_sph_ is typically 5–10%, except for cases where absorption is strong such as copper, for which the uncertainty is as high as 20%. The purity of the materials is generally unknown since they were on hand from other experiments. The materials of known purity in mass fraction are Al 99.99%, Cu 99.999% and Nb 99.99%.

**Figure 14:**
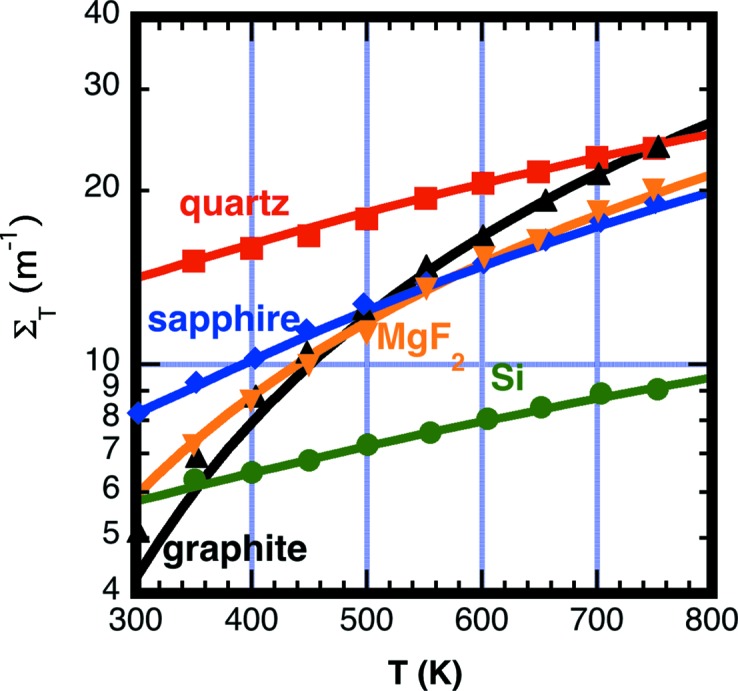
A plot showing the measured (symbols) and fitted (lines) total scattering cross section Σ_T_ according to equation (21) using values in Table 4 ▸ for Si (green), sapphire (blue), graphite (black), quartz (red) and MgF_2_ (orange). In the fitting of data, only the vertical scale to model is adjusted using parameter *f*_sph_.

Fig. 14 ▸ shows that, in general, the temperature dependence of the total cross section fits well to equation (21) for Si, sapphire, C (graphite), quartz and MgF_2_. The model underestimates the cross section for both Be and C near room temperature. The fitted values *f*_sph_ ranged from 0.85 for graphite to 2.1 for Nb, with an average value of 1.3. The variability and size of *f*_sph_ is larger than that expected from the size of the interference term obtained by Binder; his calculations predict *f*_sph_ = 1.15 for aluminium, for example. For materials not listed in Table 4 ▸, the total cross section can be reasonably approximated at long wavelengths, where Bragg and multiphonon scattering do not contribute, by using (21) if the Debye temperature is known and the mean value of *f*_sph_ = 1.3.

Of higher interest for SANS measurements is the ability to estimate the temperature-dependent background scattering from phonons in the small-angle range. As is the case for liquids and gasses, we assume the scattering is quasi-isotropic using the relation (13). These samples have small lateral dimensions compared to the mean scattering distance (1/Σ_sph_), so the fraction of scattering from multiple events is small.

**Figure 15:**
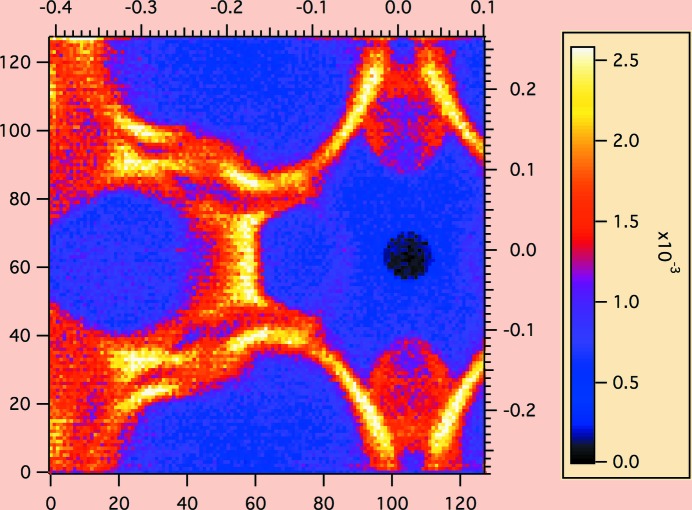
Inelastic scattering from a silicon single crystal, with the beam direction aligned along the [220] crystal direction and incident neutron wavelength λ_0_ = 8 Å. The beam was pulsed with a disc chopper. Events were recorded in time-of-flight mode, and all neutron events with time channels corresponding to quasi-elastic scattering were removed. The left and lower axes indicate the pixel position on the 128 × 128 two-dimensional detector. The upper and right axes give the *q* value in Å^−1^. The color scale gives the absolute scale of the intensity in units of cm^−1^ sr^−1^. The cross section was not corrected for the wavelength dependence of the ^3^He detector. Note the rich structure provided by the optical phonon modes at larger *q*. The inelastic scattering cross section for *q* < 0.1 Å^−1^, within the typical range for SANS measurements, was nearly flat and appreciable in intensity at *I*(*q*) ≃ 5 × 10^−4^ cm^−1^ sr^−1^.

**Figure 16:**
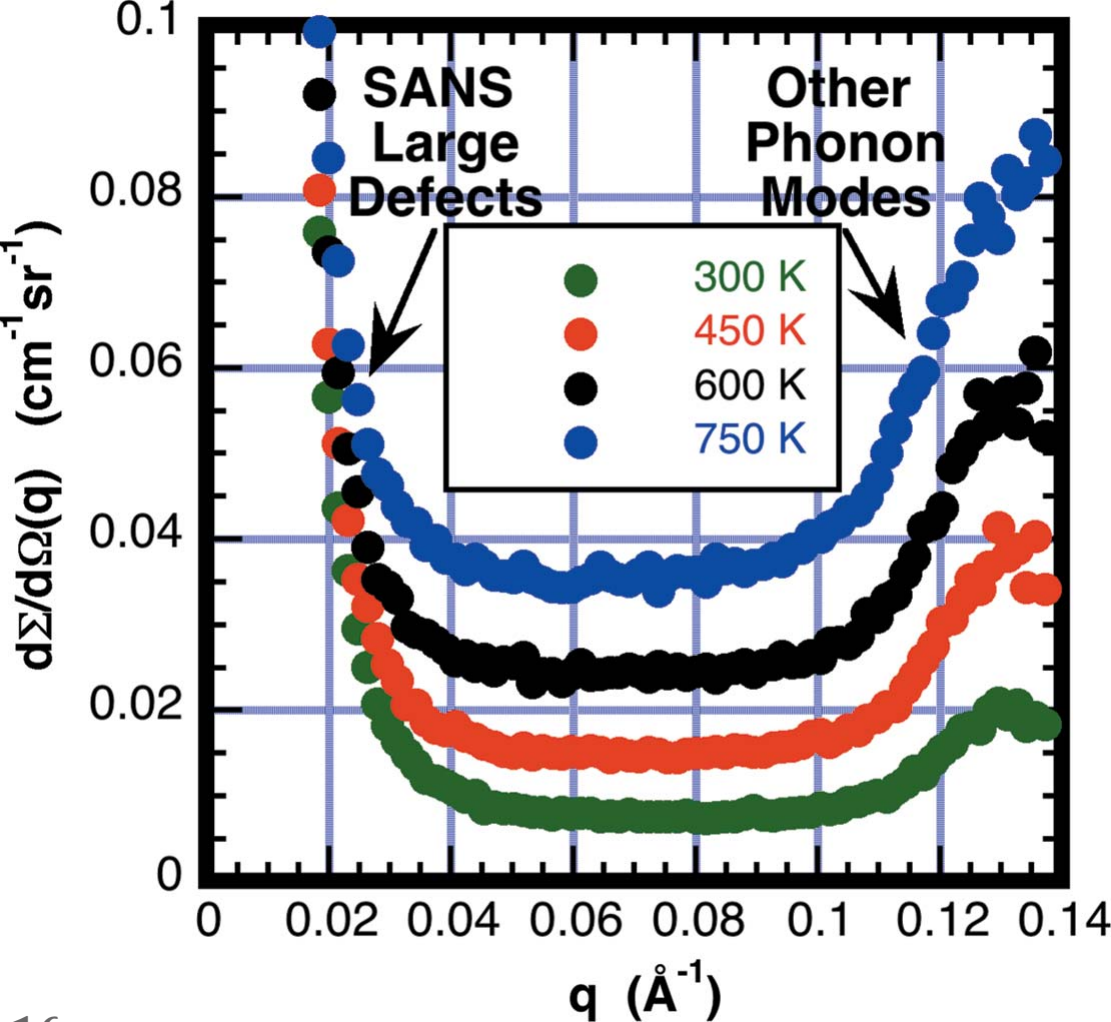
A plot showing the measured SANS scattering from filter-grade graphite as a function of temperature. The upturn for *q* < 0.04 Å^−1^, which is insensitive to temperature, is likely to originate from SANS from large defects such as voids. The scattering peak at *q* = 0.13 Å^−1^, which increases with temperature, is probably from a phonon mode. The rather flat scattering over the range 0.05 < *q* < 0.09 Å^−1^ is attributed to single-phonon scattering from transverse modes. The data shown in Fig. 14 ▸ for graphite are obtained by taking the average value over the *q* range 0.05 < *q* < 0.09 Å^−1^.

The phonon scattering is known to be non-isotropic. The inelastic scattering from a silicon single crystal, with beam direction aligned along the (220) crystal direction and incident neutron wavelength λ_0_ = 8 Å, was measured using TOF and is shown in Fig. 15 ▸. The inelastic scattering cross section for *q* < 0.1 Å^−1^, within the typical range for SANS measurements, is nearly flat. The relatively flat scattering near *q* = 0 is used in determining *G*_D_ *G*_θ_ from (13). An example of such a fit is the data from graphite shown in Fig. 16 ▸. The scattering upturn near *q* = 0 is probably due to SAS from pores or other defects. The upturn at large *q* is probably from optical phonons. The fit is restricted to the flat region 0.05 ≤ *q* ≤ 0.09 Å^−1^.

The *G*_D_ *G*_θ_ values in Table 4 ▸ were obtained using equation (13) with scattering data taken at λ_0_ = 10 Å and *L*_2_ = 2 m. Only the part of the *q* range that exhibits a flat response is included in the fit, as described for graphite in Fig. 16 ▸. Some of the single crystals exhibited a flat response over the entire *q* range, in which case all the data were included. Reasonable fits of the temperature dependence of the flat background are obtained for all materials, resulting in the values for *G*_D_ *G*_θ_ in Table 4 ▸, using (13) to approximate the forward scattering contribution.

**Figure 17:**
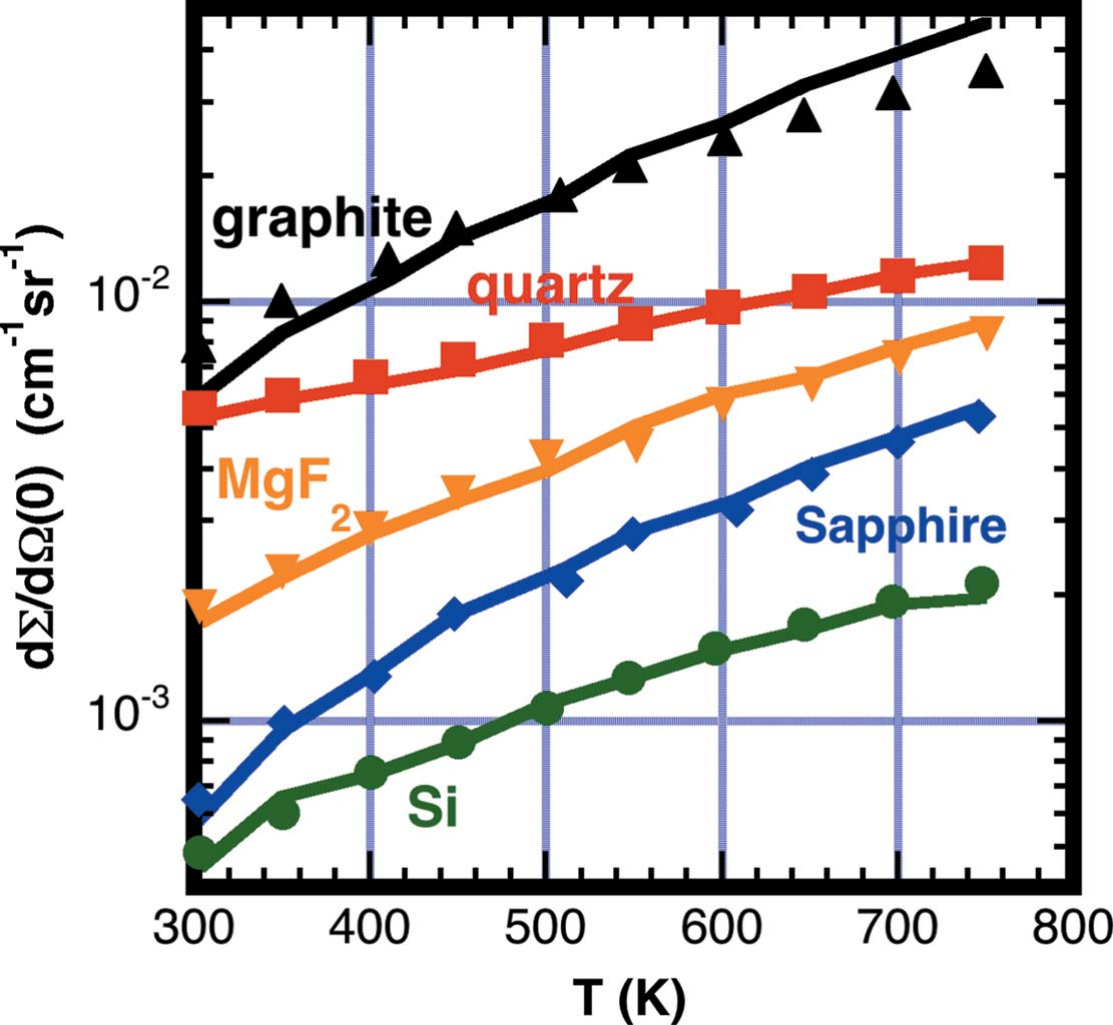
A plot showing the measured values (symbols) and values obtained from equation (13) using the sample transmission fitted values for *G*_D_ *G*_θ_ listed in Table 4 ▸ (lines) of the forward scattering cross section dΣ/dΩ(0) for Si (green), sapphire (blue), graphite (black), quartz (red) and MgF_2_ (orange).

Fig. 17 ▸ shows the fit of the measured forward scattering dΣ/dΩ(0) *versus* temperature *T* for the materials Si, sapphire, graphite, quartz and MgF_2_. Quartz also has an additional temperature-independent coherent component to the cross section Σ_coh_ = 8.4 (5) m^−1^ at λ_0_ = 10 Å owing to its glass structure. Some materials not included here may also have additional coherent background from Laue diffraction (Glinka, 2011 ▸). A material such as α brass having interstitial or substitutional site alloying is an example where strong coherent background from Laue diffraction is expected.

#### 4.3.3. Time of flight   

**Figure 18:**
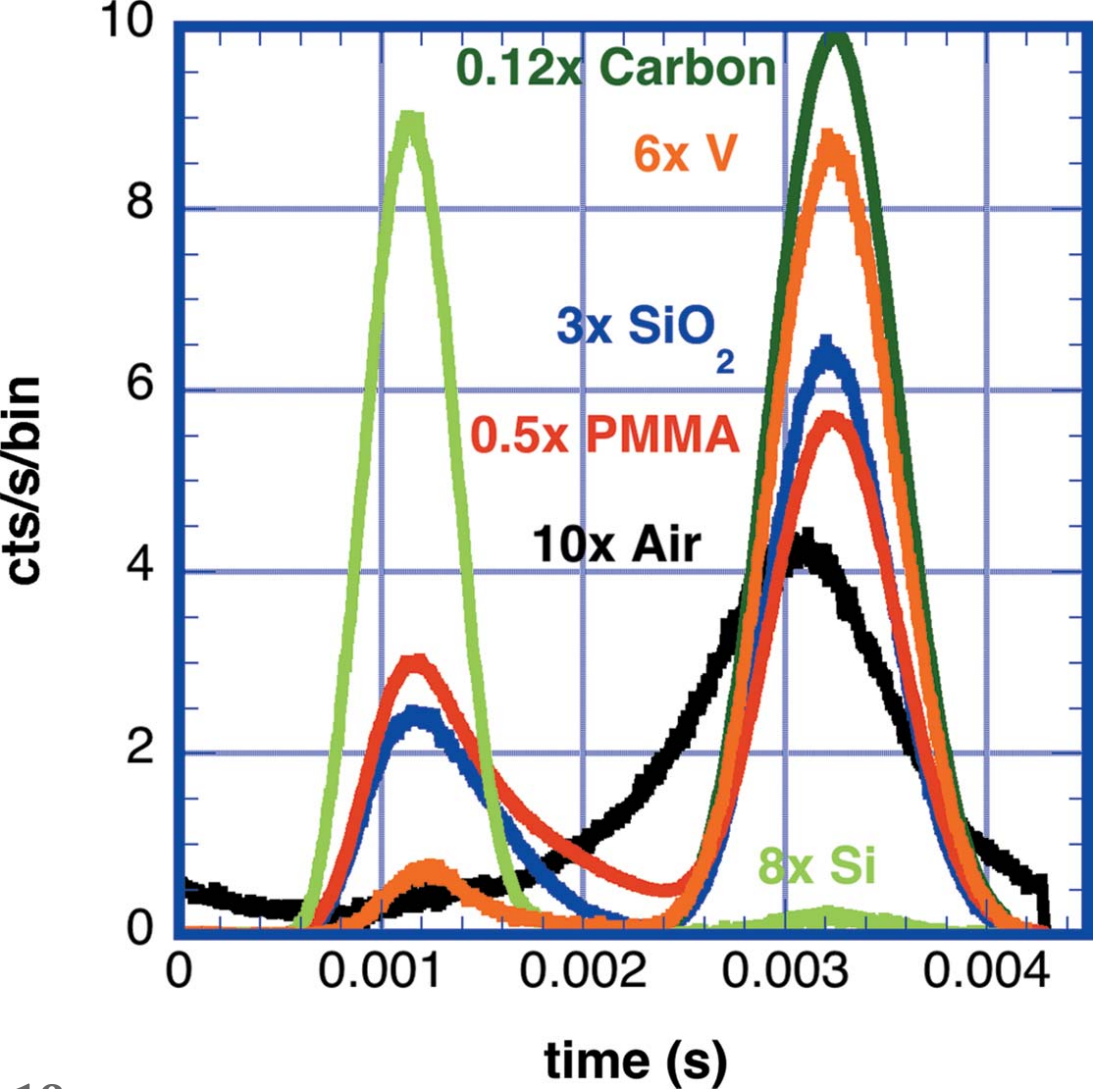
A plot of TOF data taken with a ^3^He detector corrected for background with *L*_2_ = 1.6 m and λ_0_ = 8 Å for air (black), quartz (blue), glassy carbon (dark green), methacrylate (red), a silicon (220) single crystal (lime green) and a vanadium single crystal (orange). The elastic scattering peak occurs at *t* = 0.0032 s. Note that the mean sample-to-detector distance for air was slightly shorter at *L*_2_ = 1.5 m. The data are binned into 1000 time channels on the horizontal axis. The vertical axis shows the number of counts per second per bin, rescaled by values indicated in the legend. The count rates were not corrected for the wavelength dependence of the ^3^He detector efficiency.

Additional TOF measurements were made on selected materials with a 16.7% duty cycle disc chopper. The ^3^He detector is as described in Table 2 ▸. The chopper was located upstream of a sample chamber that was evacuated to reduce the contribution from air scattering. The chopper-to-detector distance was 1.6 m. Fig. 18 ▸ shows the obtained scattered time spectra from air, quartz, glassy carbon, PMMA, Si and V. The inelastic scattering appears as a broad peak at *t* = 0.0012 s, while quasi-elastic scattering appears in a peak located at 0.0032 s. Note that the spectra are not corrected for the wavelength dependence of the ^3^He detector efficiency, since the large duty cycle smears the wavelengths, particularly at short wavelengths. Fig. 11 ▸ predicts that the inelastic peak amplitude would increase by a factor of two if corrected. The glassy carbon sample has strong SAS from porosity, so nearly all scattering is elastic. Similarly, the scattering from vanadium is dominated by elastic incoherent scattering. Scattering from silicon in this angular range is dominated by phonon scattering. Scattering from quartz and PMMA have significant contributions from both inelastic and elastic scattering processes. Scattering from air is elastic, but with a broadening caused by the velocity spectrum and random direction of molecules in a gas.

Table 5 ▸ provides the fractions of inelastic scattering *f*_in_ obtained from spectra shown in Fig. 18 ▸. At room temperature, all of the solid materials exhibited measurable inelastic scattering except for glassy carbon. Incoherent scattering from vanadium single crystals is used as a primary absolute intensity calibration of SANS instruments (Wignall & Bates, 1987 ▸). The calculated absolute cross section is an underestimate because it does not include the inelastic fraction. For the vanadium single crystal, 6.2% of the detected events are inelastically scattered. The size of the inelastic correction to the calibration also depends upon wavelength and detector efficiency.

**Figure 19:**
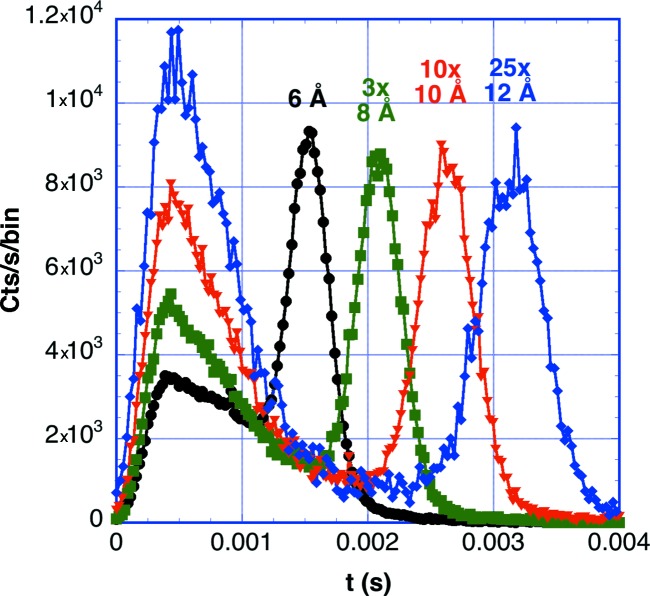
A plot of TOF data taken with a ^3^He detector corrected for air background taken at *L*_2_ = 2.0 m for a 1 mm-thick H_2_O sample at wavelengths 6 Å (black), 8 Å (green), 10 Å (red) and 12 Å (blue). The vertical axis has the number of counts/bin, rescaled by values indicated in the figure captions. The counts were not corrected for the wavelength dependence of the ^3^He detector efficiency.

Additional TOF measurements were made as a function of wavelength at λ_0_ = 6, 8, 10, 12 and 15 Å on 1 mm-thick H_2_O and 4 mm-thick D_2_O samples contained with windows having 3.2 mm thickness of quartz, with samples and chopper in air. The air background was subtracted using empty and beam-blocked measurements. The spectra from H_2_O are shown in Fig. 19 ▸. The spectra from D_2_O are similar, with a slightly greater inelastic contribution. The inelastic fractions with and without correction of detection efficiency are listed in Table 6 ▸. The increase in the inelastic fraction with longer wavelengths coincides with the increase in the scattering cross section for water. Ghosh & Rennie (1999 ▸) have previously shown this dependence and have reported comparable inelastic fractions. Other measurements on water show an increase in the inelastic fraction with increasing sample thickness that is caused by increases in the fraction of multiply scattering events, since a shift to other wavelengths is caused by a single inelastic interaction.

## 5. Discussion and conclusions   

Reducing the background scattering in SANS measurements improves the precision obtained in the absolute scattering cross section dΣ/dΩ(*q*) by increasing the ratio of the desired signal from coherent SAS from nanostructures in the sample to the background produced both by the sample and the instrument. This paper shows how to estimate the background from gases, liquids or solids as a function of temperature and wavelength. All materials exhibit an increase in inelastic background scattering with increasing cold neutron wavelength λ_0_ > 5 Å. The single-phonon cross section for cold neutrons is proportional to wavelength. The inelastic scattering cross section from water also shows a linear dependence upon wavelength at wavelengths λ_0_ > 10 Å. Thus, using shorter wavelengths usually improves the signal-to-noise ratio (S/N).

Note that the current analysis does not include Bragg and multiphonon scattering, which have important contributions in the thermal neutron range 1 ≤ λ_0_ < 5 Å (Freund, 1983 ▸). For single crystals, a minimum in the total scattering often occurs in the wavelength range 1 ≤ λ_0_ < 3 Å. For single-crystal and some liquid samples, reducing the wavelength λ_0_ from 5 to 2 Å can produce a further small improvement in S/N. On the other hand, to avoid double Bragg scattering in crystalline materials often requires λ_0_ ≥ 5 Å. For many SANS instruments that view a cold source moderator, achieving the maximum scattering count rate usually means the use of longer-wavelength cold neutrons. The optimum choice for wavelength is therefore often a compromise between the lowest background and the highest beam current on the sample. Operating at longer wavelengths can reduce the coherent scattering from D_2_O, for example. The total cross section for D_2_O has a local minimum at λ_0_ ≃ 9 Å (Mattes & Keinert, 2005 ▸). A similar local minimum is found in the total cross section data for quartz.

Note that the inelastic fractions *f*_in_ presented in this paper are as measured using the ^3^He detector as described in Table 2 ▸. The accuracy of the measured fractions depends upon correctly determining the wavelength dependence of the detector efficiency. Measurements with a higher-efficiency scintillation detector would minimize this error. There is increased uncertainty in the wavelengths at short flight times owing to the large chopper duty cycle and the overlap of the inelastic and elastic spectra. The measured value for *G*_D_ *G*_θ_ in Table 4 ▸ would also increase. For the same reason, using scattering from H_2_O or V as an absolute scattering standard requires a detector-dependent correction.

The inelastic fraction of the background also depends upon the temperature of the measurement. Cryogenic cooling of components can largely eliminate this source, but liquid samples can normally not be frozen without deleterious effects. Many experiments are performed at multiple temperatures where the inelastic background is changing. In some cases the background is determined at a different temperature from the sample. Samples that have magnetic scattering at low temperature often have the background subtracted at a higher temperature where the coherent magnetic scattering is eliminated by a phase transition. But, the higher temperature also increases the inelastic background, causing an improper correction.

If the background is dominated by the sample, the use of thinner samples that minimize multiple scattering provides a small advantage. Typically, textbooks recommend that the sample thickness be chosen that maximizes the scattering signal, which corresponds to a sample thickness *d*_S_ = 1/Σ_T_. Where absorption is the dominant attenuation process in the sample or if the background signal from the sample is small compared to the instrument background, this is true. But, if the scattering from the sample is dominant, the background will increase as 1/*T*_S,S_ as in (13). The optimized sample thickness factoring the reduced S/N now becomes *d*_S_ = 0.48/Σ_T_. For an infinite slab geometry where multiple scattering enhances the forward scattering, the optimum thickness is reduced further to *d*_S_ = 0.35/Σ_T_. Most water samples fall in the last case. For H_2_O at λ_0_ = 5 Å the optimum sample thickness is *d*_S_ = 0.5 mm, whereas for D_2_O at λ_0_ = 9 Å the optimal *d*_S_ = 5 mm. To enhance the S/N the sample should have the highest concentration that does not produce aggregation effects. For materials such as biological molecules where the amount of sample is limited by cost or preparation time, the sample thickness may be further constrained by the amount of material available.

The S/N can be increased by roughly a factor two by using TOF to exclude inelastic events for water samples. To obtain good separation of the inelastic and elastic events, a 25% duty cycle chopper can be used with λ_0_ = 8 Å neutrons. If counting statistics dominate the error in the measurement, the factor of two improvement in S/N is canceled by the factor of four reduction in count rate caused by the duty cycle of the chopper.

Alternatively, a polycrystalline Be filter can be placed between the sample and the detector. A 5 cm-thick Be filter cryogenically cooled to *T* = 77 K will transmit 2.5% of λ_0_ = 1 Å neutrons that are inelastically scattered but transmit 97.6% of elastically scattered neutrons at λ_0_ = 5 Å. The filter is best located midway between sample and detector, with a central hole to allow the unscattered beam to pass through. Broadening of the beam by refraction from micrometre-sized voids or oxide inclusions from porosity found in Be filters has been measured (Glinka *et al.*, 1986 ▸). At λ_0_ = 5 Å, the FWHM of the broadening for a 5 cm-thick filter of fine-grain hot-pressed Be material is 0.003 rad (0.2°). The scattering produces wings on the effective point spread function that probably produce smearing effects similar to that observed from dome scattering shown in Fig. 6 ▸. Such a filter could increase the S/N by a factor of two for water samples without reducing the count rate appreciably. However, the design of a cryogenic filter located inside the vacuum vessel that does not interfere with detector travel would be difficult on many existing SANS instruments.

A further factor of two increase in S/N can be achieved in principle by using full polarization analysis of the spin scattering to exclude spin-flip scattering produced by incoherent scattering from water (Gentile *et al.*, 2000 ▸; Babcock *et al.*, 2013 ▸). The instrument needs to polarize the beam before the sample and an analyzer is needed to determine the spin after the sample. All SAS events are non-spin flip. Incoherent scattering from H or D has 1/3 probability of non-spin flip and 2/3 probability of spin flip. Water samples typically have a large component of multiply scattered events. However, in the limit of a large number of interactions, the average spin flip from water is typically very close to 1/2. See Babcock *et al.* (2013 ▸) for further discussion. For adequate performance of the ^3^He spin transmission analyzer the inelastic events must be removed separately. For full spin analysis to reduce the error in the measurement if dominated by counting statistics, the combined beam attenuation of the polarizer and analyzer must be less than a factor of four. Ideally, beam polarization reduces the beam current by a factor of two.

Some improvements over our current operation can be readily adopted. Consider a hypothetical low-concentration biological molecule sample in D_2_O. The sample concentration is made as high as feasible without producing aggregation. The isotopic purity of the D_2_O is 99.9%. Some contamination with H is produced with sample preparations in humid air. Here the H/D ratio averaged throughout the sample, and including the biological material, is assumed to be 0.005. The forward scattering from H_2_O is 20 times as strong as that from D_2_O. The sample temperature is maintained at *T* = 283 K, to minimize denaturing but not to freeze the sample. The amount of material available is limited, so the sample thickness is restricted to *d*_S_ = 1 mm. A neutron wavelength of 6 Å is used. The sample chamber is used with a 0.15 m-long air path, with a 6.4 mm-thick silicon window separating the sample chamber from the vacuum in the detector vessel and a 3.2 mm-thick sapphire window separating the sample chamber and collimation vessel from air. The sample cell uses quartz windows having 3.2 mm thickness. From data provided in this paper, the fraction of the background produced by each component is D_2_O 57%, H_2_O 6%, air 19%, quartz 14% and Si + sapphire 4%. Simply evacuating the sample chamber will reduce the background by 19%. Replacing quartz with lower-background single-crystal sapphire windows on the sample cell will reduce the background a further 13%. Such improvements can be easily accommodated.

## Figures and Tables

**Table 1:** Material constants used to calculate the scattering cross-section parameters for six typical gases *A*_e_ is the effective mass of the molecule in atomic mass units. The bound scattering _*i*,b_ and absorption _*i*,a_ cross sections in barns are obtained from Sears (1986 ▸, 1992 ▸). The mole fractions *X_i_* listed are for air with water content to produce condensation at *T* = 283K.

Molecule	*A*_e_ (u)	_*i*,b_ (b)	_*i*,a_ (b)	*X_i_*
N_2_	9	23.02 (22)	3.80 (6)	0.773
O_2_	10	8.464 (12)	0.00038 (4)	0.206
H_2_O	2	168.27 (12)	0.6654 (14)	0.012
Ar	39.9	0.683 (4)	0.675 (9)	0.009
He	4.0	1.34 (2)	0.00747 (1)	
CH_4_	4	333.6 (2)	1.334 (3)	

**Table 2:** Parameters (thickness of the window *d*_w_ and active depth *d*_d_ of the detector and the absorption cross sections) used in equation (16) to calculate the wavelength-dependent detection efficiency for two different neutron detectors, using ^3^He gas or ^6^Li in a glass scintillator

Variable	^3^He gas	^6^Li scintillator
*d*_w_ (cm)	0.8	0.4
_a,w_/ (cm^1^ ^1^)	7.79 10^3^	7.79 10^3^
*d*_d_ (cm)	2.5	0.2
_a,d_/ (cm^1^ ^1^)	0.146	23

**Table 3:** Forward scattering *I*_f_(0) from coherent scattering from thermal fluctuations in density as calculated using equation (18) for both neutrons and X-rays (values in parentheses) The compressibility of H_2_O is obtained from Orthaber *et al.* (2000 ▸) and that of quartz from Emerson (1937 ▸). Scattering from water fluctuations is used as a primary absolute standard in SAXS measurements. For SANS, the fluctuations contribute 10% of the signal for D_2_O and negligible fractions of the total *I*(0) for H_2_O or quartz.

Material	Compressibility _T_ (Pa^1^)	Scattering length density _l_ (^2^)	Forward scattering *I*_f_(0) (cm^1^sr^1^)
H_2_O	4.58 10^10^	0.56 10^6^	5.9 10^5^
		(9.31 10^6^)	(0.0163)
D_2_O	4.58 10^10^	6.37 10^6^	7.6 10^3^
Quartz	9.8 10^12^	3.48 10^6^	4.9 10^5^
		(18.9 10^6^)	(1.4 10^3^)

**Table 4:** Material parameters used in fitting the temperature dependence of the total scattering cross section _T_ and the forward scattering cross section d/d(0) Parameters: *d*_S_ is the sample thickness, _m_ is the mass density, *M*_e_ is the effective molar mass, _D_ is the material Debye temperature, _a_ is the absorption cross section and _bat_ is the bound atom cross section (Barker *et al.*, 2008 ▸). The vertical scale factors *f*_sph_ and *G*_D_ *G* were obtained by fitting _T_ and d/d(0) to equations (21) and (13), respectively. Measurements were made with a ^3^He detector. The quantity *G*_D_ depends strongly on detector efficiency.

Material	*d*_S_ (cm)	_m_ (gcm^3^)	*M*_e_ (gmol^1^)	_D_ (K)	_a_/_0_ (m^1^ ^1^)	_bat_ (m^1^)	*f*_sph_	_inc_ (m^1^)	*G*_D_ *G*
Al	3.0	2.70	26.98	428	0.774	9.06	1.4		0.58 (2)
Sapphire	5.4	3.98	17.35	1040	0.605	36.91	1.1		0.42 (2)
Be	5.8	1.848	9.01	1440	0.0522	94.24	1.5		0.3 (1)
C (gr)	4.7	2.26	12.01	1050	0.0221	62.9	0.9		1.3 (2)
Cu	0.58	8.94	63.55	343	17.7	68.0	1.0	4.3	1.0 (2)
Ge	0.96	5.32	72.61	374	5.3	37.9	1.3	0.80	0.7 (2)
MgF_2_	4.0	3.148	20.45	610	0.139	35.74	1.2		0.39 (3)
MgO	3.9	3.58	19.04	938	0.188	42.48	1.5		0.63 (6)
Nb	0.8	8.57	92.91	275	3.55	34.7	2.1		0.39 (6)
Si	5.4	2.33	28.09	645	0.475	10.82	1.4		0.48 (6)
Quartz	5.1	2.20	17.54	580	0.210	23.44	0.9	8.4	0.41 (3)

**Table 5:** The inelastic fraction of scattering for materials taken from TOF data with a 16.7% duty cycle chopper and a ^3^He detector, with _0_ = 8 and a sample-to-detector distance of *L*_2_ = 1.6m The inelastic fraction is estimated from the fraction of the total counts found within the inelastic peak as shown in Fig. 18 ▸. Data in the last column are corrected for the wavelength dependence of the detector efficiency for the ^3^He detector found in Fig. 10 ▸. All measurements were made at room temperature.

Sample	*d*_S_ (cm)	_D_ *f*_in_	*f*_in_
Quartz	1.8	0.28(1)	0.38(2)
Al	2.3	0.66(2)	0.76(3)
Glassy carbon	0.10	0.0008(3)	0.0012(5)
PMMA	0.14	0.35(2)	0.45(3)
Nb	0.38	0.45(2)	0.56(3)
Si (220)	7.6	0.973(4)	0.993(6)
V	0.35	0.062(4)	0.090(6)

**Table 6:** The inelastic fraction of scattering for 1mm-thick H_2_O and 4mm-thick D_2_O at several wavelengths taken from TOF data with 8.3% duty cycle chopper measured with a ^3^He detector with the sample-to-detector distance of *L_2_* = 2m obtained by integrating spectra as shown in Fig.19 ▸ All measurements were made at room temperature. Scattering from 3.2mm of quartz from the sample cell is included, and air background is subtracted. Data are also corrected for the wavelength dependence of the detector efficiency, which increases the fractions considerably. Note, for all samples the inelastic fraction increases with increasing wavelength. The inelastic fraction also increases with increasing sample thickness as a result of multiple scattering.

	H_2_O, *d*_S_ = 1mm		D_2_O, *d*_S_ = 4mm	
_0_ ()	_D_ *f*_in_	*f*_in_	_D_ *f*_in_	*f*_in_
6	0.45 (2)	0.63 (3)	0.57 (2)	0.69 (3)
8	0.53 (2)	0.67 (3)	0.61 (2)	0.72 (3)
10	0.57 (2)	0.72 (3)	0.64 (2)	0.75 (3)
12	0.63 (2)	0.74 (3)	0.65 (2)	0.79 (3)
15	0.66 (2)	0.82 (3)	0.73 (2)	0.87 (3)
